# Oral health-related quality of life in patients with periodontitis: a systematic review and meta-analysis

**DOI:** 10.3389/froh.2025.1503829

**Published:** 2025-05-26

**Authors:** Joanna Slowik, Anna Panasiuk, Marcin Kaczor, Marcin Wnuk

**Affiliations:** ^1^ Department of Periodontology, Preventive Dentistry and Oral Pathology, Faculty of Medicine, Jagiellonian University Medical College, Krakow, Poland; ^2^ Aestimo s.c., Krakow, Poland; ^3^ Jagiellonian University Medical College, Kraków, Poland; ^4^ Department of Neurology, Jagiellonian University Medical College, Krakow, Poland

**Keywords:** oral health-related quality of life, periodontitis, systematic review, meta-analysis, OHIP-14

## Abstract

**Objective:**

This study aimed to comprehensively evaluate the impact of periodontitis on Oral Health-Related Quality of Life (OHRQoL) using the OHIP-14 questionnaire. A quantitative meta-analysis was conducted to estimate the average effect size, taking into account the characteristics of periodontitis and the features of control groups. Additionally, associations between OHRQoL and periodontitis were explored based on participant demographics and clinical factors.

**Methods:**

We systematically searched the PubMed, Embase, and Scopus databases up to March 8, 2024. Studies included in the analysis assessed OHRQoL in patients with periodontitis (exposed group) compared to non-periodontitis individuals (non-exposed control group). A valid periodontitis diagnosis required Clinical Attachment Loss (CAL) and Pocket Probing Depth (PPD) assessments during full-mouth clinical examinations. The choice of meta-analysis model was based on an assessment of heterogeneity. The quality of the included studies was assessed using the tool developed by The National Heart, Lung, and Blood Institute (*N*HLBI).

**Results:**

Nine studies, encompassing 2,287 individuals, met the inclusion criteria. Periodontitis significantly affected the mean OHIP-14 total scores compared to controls [Weighted Mean Differences WMD random = 6.11 (95% CI: 4.23, 7.99), *p* < 0.0001], with substantial heterogeneity. Subgroup analysis did not reveal significant regional variations. Restricting the analysis to studies using the American Academy of Periodontology/European Federation of Periodontology consensus definition from 2017 yielded similar results. The negative impact of periodontitis on OHRQoL was associated with disease severity and female sex but was not influenced by the region or age of the study participants.

**Conclusions:**

Our findings confirm that periodontitis significantly impairs OHRQoL, with potential associations related to disease severity and sex. However, the limited availability of studies with matched control groups and poor data reporting quality constrains a more comprehensive assessment.

## 1 Introduction

Periodontitis is a common inflammatory disease that detrimentally affects both the soft and hard tissues surrounding the teeth (1). This condition is caused by imbalance between microbes in dental plaque and the inflammatory host responses (2). Gingivitis, that precedes periodontitis in susceptible individuals, is characterized by reversible inflammation of the gingiva, whereas periodontitis causes irreversible damage to the periodontal tissues, manifesting as apical migration of the epithelial attachment, clinical attachment loss (CAL) of the periodontal ligament, loss of alveolar bone, and eventually tooth mobility and possible tooth loss (3). Therefore, periodontitis accounts for a significant proportion of edentulism and masticatory dysfunction, incurs substantial dental care costs, and plausibly negatively impacts general health (4–7). The World Health Organization (WHO) defines severe periodontal disease as one of the five main oral conditions of public health relevance, that is also strongly associated with diabetes mellitus and potentially with cardiovascular disease (8). Its global prevalence is estimated at about 19% in individuals aged over 15 years, that translates into more than 1 billion cases worldwide (8).

Despite the serious long-term consequences, periodontitis may initially go unnoticed, without symptoms troublesome to the patient (9). On the other hand, advanced periodontitis may cause symptoms such as bleeding gums, mobile teeth, pain on chewing, halitosis, and aesthetic problems (4, 10). This implies that periodontitis can have a substantial impact on quality of life, specifically on the Oral Health-Related Quality of Life (OHRQoL), that is a multidimensional concept describing the impact of oral health on a person's daily functioning, psychological well-being, and social life (11–14).

As a latent construct, OHRQoL cannot be directly observed. In both clinical practice and scientific research, a variety of tools and indicators are employed to measure it (15). Among various tools assessing OHRQoL, the most commonly used is Oral Health Impact Profile-14 (OHIP-14) (15) which represents a shortened version of the Oral Health Impact Profile (OHIP). In the full 49-item version, OHIP was developed by Slade and Spencer in 1994 (16), and the shortened 14-item version was introduced in 1997 (17). The 7 subscales included in the OHIP-14 encompass difficulties related to functional limitation, physical pain, psychological discomfort, physical disability, psychological disability, social disability, and handicap (17). Several studies have questioned the suitability of the original seven-factor structure, supporting alternative models, including various multidimensional structures, most often comprising three (18–21) or four (22) distinct factors. Conversely, other research suggests that the OHIP-14 operates primarily as a unidimensional scale, measuring a single underlying construct commonly interpreted as general “oral ill-health” or the overall impact on oral health-related quality of life (OHRQoL) (23, 24). Consequently, the empirical factorial structure of the OHIP-14 remains a subject of considerable debate, with a recent review of OHIP versions suggesting that a 4-dimensional structure (comprising Oral Function, Orofacial Pain, Orofacial Appearance, and Psychosocial Impact) is a more valid and parsimonious model (19). Nonetheless, the OHIP-14 is recognized as a convenient, short-scale instrument, consistently found to be reliable and valid for assessing OHRQoL across diverse populations and linguistic contexts (25–29). With regard to reliability, research generally reports acceptable to excellent internal consistency for the overall OHIP-14 scale, with Cronbach's alpha values frequently exceeding 0.80 (25–29).

The impact of periodontitis on OHRQoL has been investigated in previous systematic reviews (15, 30–33). However, the applicability of their results is hindered due to the inclusion of primary studies that have relied on outdated definitions and/or assessment methods for periodontitis (15, 30, 32). The most recent of the identified systematic reviews, which assessed the impact of periodontitis on quality of life (QoL) measured with OHIP-14 in the adult population aged 18–70 years, provided evidence of a significant relationship between periodontitis and oral health-related quality of life (OHRQoL), as well as the association of risk poor OHRQoL with the stage of periodontitis (31). However, this review was based on a set of heterogeneous studies published between 2008 and 2020 (4). During this period, significant changes occurred in the recommendations regarding the definition as well as the standards for assessing this condition in epidemiological research. None of the studies assessed in the mentioned review were based on the latest consensus case definition established during the 2017 World Workshop on the Classification of Periodontal and Peri-Implant Diseases and Conditions (4). Additionally, some of the included studies had significant limitations, such as assessing fewer than four sites/teeth (34–36), using partial mouth examination (37), defining a group with periodontitis solely based on radiographic assessment (38), or the simultaneous presence of caries in all patients with periodontitis (39). Other systematic reviews published in recent years have focused on a narrow population of elderly individuals (33) or assessed periodontal diseases as a whole, without distinguishing periodontitis (40). Therefore, there is a need for a reliable estimation of the impact of periodontitis on OHRQoL in a broad population of adult patients, taking into account studies in which periodontitis was diagnosed according to the current definition.

Thus, our main objective was to conduct a comprehensive quantitative assessment of the impact of periodontitis on OHRQoL, conceptualized as the overall burden of oral health impacts on the individual, through a meta-analysis of available numerical data, based on the following clinical question: “How does periodontitis, as defined by probing pocket depth and clinical attachment loss determined by a full-mouth clinical examination, affect OHRQoL in adults compared to those with clinically healthy periodontium or gingivitis, as measured by the OHIP-14 in observational studies?” A secondary objective was to explore how potential associations between periodontitis and OHRQoL, as assessed using OHIP-14, were influenced by demographic and clinical characteristics of study participants, including variability in definitions and characteristics of periodontitis and the features of the control groups.

## 2 Methods

### 2.1 Protocol

No protocol was prospectively registered for this review.

### 2.2 Search strategy and selection criteria

A comprehensive search was conducted in PubMed, Embase, and Scopus databases, covering literature up to March 8, 2024. The search strategies combined terms defining periodontitis with OHRQoL or OHIP-related keywords using the boolean operator “AND.” No additional filters were applied. Detailed search strategies were presented in Supplementary Tables S1–S3. Reference lists of previously published reviews with a similar scope, as well as primary studies meeting the inclusion criteria, were examined to identify any potentially overlooked studies.

The eligibility criteria for this systematic review and meta-analysis were defined according to the PECOS (Population, Exposure, Comparator, Outcome, Study) scheme. We included studies assessing the impact of periodontitis on OHRQoL in the general adult population (≥18 years old). Cohort, case-control, or cross-sectional studies that assessed OHRQoL with the OHIP-14 scale among patients diagnosed with periodontitis (exposed group) compared to non-periodontitis control groups (non-exposed group, i.e., clinically healthy periodontium, gingivitis, or a combination of patients with these conditions) were eligible for inclusion. The diagnosis of periodontitis was considered valid if based on at least Clinical Attachment Loss (CAL) and Pocket Probing Depth (PPD) assessed in a full-mouth clinical examination, with at least four measurement points per tooth. The primary outcome was OHRQoL, measured by the OHIP-14. In line with our objective to conduct a quantitative meta-analysis, we included only these studies that adequately summarized the outcomes of the OHRQoL assessments. At least one of the following measures, or data that allowed for its calculation, had to be reported, as defined by Slade and colleagues: (i) severity of impacts, i.e., the mean OHIP-14 total score (the sum of ordinal responses) along with the standard deviation (SD); (ii) prevalence of impacts, i.e., the percentage of study participants reporting “fairly often” or “very often” on one or more items; (iii) extent of impacts, i.e., the mean (SD) total number of items reported “fairly often” or “very often” by study participants (41). Studies published in English or Polish were eligible.

We excluded studies conducted in children or adolescents. To avoid confounding due to comorbidities, we did not include studies where populations were selected based on any comorbidities (e.g., diabetes) or specific physiological conditions (e.g., pregnancy). Moreover, to ensure that exposure groups truly included patients with periodontitis, we excluded studies using the Community Periodontal Index of Treatment Needs (CPITN), Community Periodontal Index (CPI), or Periodontal Screening and Recording/Periodontal Screening Index (PSR/PSI), or those based solely on CAL, PPD, or radiographic assessment. Non-exposed groups could not include patients with periodontitis; therefore, control groups that included patients with mild or localized periodontitis were considered inadequate. We also did not include studies where the OHRQoL of patients with periodontitis was assessed as a measure of the effectiveness of new treatment interventions (i.e., interventional studies), as our aim was to evaluate OHRQoL in a cross-sectional, real-world patient population. For a more detailed description of the eligibility criteria, please refer to Supplementary Table S4.

### 2.3 Data collection

Two independent reviewers (A.P. and M.K.) screened titles and abstracts, and subsequently full-text articles, to determine their suitability for final inclusion. At each stage of the assessment, any discrepancies between the reviewers were discussed, with the involvement of a third researcher (J.S.), allowing a consensus decision to be reached regarding the eligibility and inclusion of all articles. One reviewer (A.P.) extracted the data (details of study design, country, eligibility criteria, baseline characteristics and predefined outcome data) to the data extraction tables in the MS Excel spreadsheets, and then it was checked by a second reviewer (M.K.). In the case of including more than one study conducted in the same country, we checked whether their populations could overlap (by verifying the region and/or names of clinics where patients were examined). Missing baseline characteristics or outcomes were calculated from other available numerical data whenever possible. If this was not feasible (i. e. no relevant outcome could be extracted or calculated), the study was excluded from the respective meta-analysis. We did not contact the original study authors to request missing data, and no data were imputed.

### 2.4 Data analysis

Two independent reviewers (A.P. and M.K.) assessed the quality of the included studies using the tool developed by The National Heart, Lung, and Blood Institute (NHLBI) for evaluating observational cohort and cross-sectional studies (42). Each study could have been rated as “Good”, “Fair”, or “Poor”. Assessment of reporting bias was planned using funnel plot and Egger's test.

To assess the difference between periodontitis and non-periodontitis subjects in continuous outcome measures (i.e., the severity of impacts and the extent of impacts), we planned to calculate Weighted Mean Differences (WMD) with 95% Confidence Intervals (CI). For the dichotomous outcome (the prevalence of impacts), the calculation of the Odds Ratio (OR) with 95% CI was intended. The meta-analysis of each summary measure was conducted provided that this outcome was evaluated in at least three studies. To explore possible causes of heterogeneity, we planned to conduct subgroup analyses based on region, periodontitis definitions, proportions of patients in different stages of periodontitis in the exposed group, and the proportion of patients with gingivitis in the control group. Furthermore, the potential association of age, sex, PPD and CAL with differences in OHRQoL was assessed in meta-regression analyses. The calculations were conducted using the IBM SPSS Statistics for Windows (Version 29.0.2.0; Armonk, NY: IBM Corp.). We selected the meta-analysis model based on the results of heterogeneity assessment. In cases of statistically significant heterogeneity (*p* < 0.1 in Cochran's *Q* test), the meta-analysis was conducted using a random-effect model; otherwise, a fixed-effects model was employed. Results of the meta-analyses were presented in forest plots, and meta-regression results were shown in a summary table and bubble plots.

The review was reported according to the PRISMA guidelines (43, 44), with the PRISMA checklist included in the Supplementary Materials (Supplementary Table S8).

## 3 Results

### 3.1 Included studies

#### 3.1.1 Study selection

**Figure 1:**
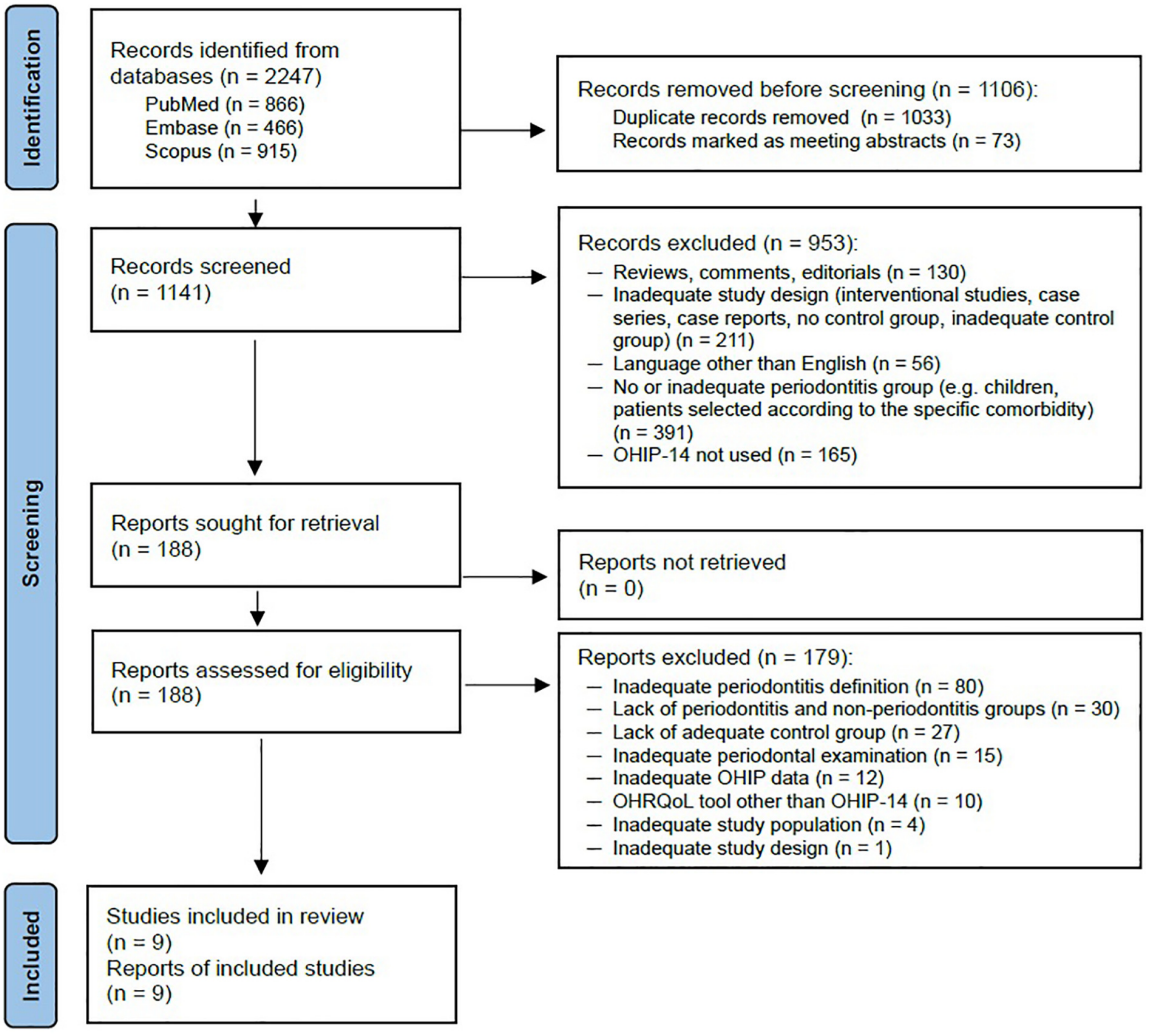
Flow chart of search results (adapted from PRISMA 2020 flow diagram) (43).

From the initial pool of 2,247 records, duplicates and conference reports were excluded, resulting in 1,141 records being screened as titles and abstracts. We considered 188 records to be potentially eligible and obtained their full texts for further consideration. We excluded 179 reports, predominantly due to the application of an inappropriate definition of periodontitis, failure to distinguish between groups with and without periodontitis within the study population, or the absence of an appropriate control group. At the full-text selection stage, the reviewers demonstrated a substantial level of agreement, as indicated by a Cohen's kappa coefficient of 0.7. Ultimately, nine publications from 9 studies, conducted in non-overlapping populations, met the inclusion criteria for the meta-analysis (see also the PRISMA flow diagram in Figure 1). Supplemental searches of the reference lists of other reviews and included publications did not yield any further studies meeting the inclusion criteria.

#### 3.1.2 Study characteristics

The characteristics of the included studies are summarized in Table 1. Out of the nine studies included, five were conducted in Asia (45–49), three in European countries (50–52), and one in South America (53). The studies were performed in non-overlapping populations, i.e., in three different regions of Turkey and—in the remaining studies—in different countries. Three studies were described as case-control design (51–53), but only in one (52) was the control group matched to the exposed group (for age and sex). The remaining six studies (45–50) were described as cross-sectional. Only four studies provided a description of the sampling method (45, 47, 50, 53), and in each, the sample was randomly selected.

**Table 1:** Characteristics of included studies.

Study, country	Country	Study period	Type of the sample (representativeness)	Sample size/power calculation?	Sampling method	Matched controls?	*N*
Al Habashneh et al. (45)	Jordan	Jan.–June 2009	Convenience (dental clinic patients)	No	Randomized	No	400
Botelho et al. (SoPHiAS substudy) (50)	Portugal	Dec. 2018–Apr. 2019^a^	Population-based (representative for a region, but age subset analyzed)	No^c^	Randomized, geographically stratified	No	592
Cataldo et al. (53)	Brazil	Sept.–Dec. 2019	Convenience (dental clinic patients)	Yes	Randomized	No	100
Dikilitaş et al. (46)	Turkey	Jan.–Sept. 2020	Convenience (oral and maxillofacial radiology clinic patients)	Yes	Not described	No	166
Eroğlu et al. (47)	Turkey	Oct.–Dec. 2022	Convenience (dental clinic patients)	Yes	Randomized	No	124
Fuller et al. (51)	UK	Not reported	Convenience (dental clinic patients)	No	Not described	No	471
Mishra et al. (48)	India	Apr. 2021–July 2022	Convenience (dental clinic patients)	Yes	Not described	No	100
Santonocito et al. (52)	Italy	Not reported	Convenience (dental clinic patients^b^)	Yes	Not described	Yes (age and sex)	111
Ustaoğlu et al. (49)	Turkey	Jan.–June 2018	Convenience (dental clinic patients)	Yes	Not described	No	223

^a^ Description in another publication (54).

^b^ The periodontitis cases were recruited from the dental clinic but the source population for the control group is unknown.

^c^ In another publication sample size calculation was provided for the SoPHiAS study (55), but not for the age subgroup analyzed in the Botelho et al. (50).

#### 3.1.3 Participants characteristics

Generally, the studies were conducted on convenience samples drawn from the patient pool of dental clinics. Only one study (the Portuguese SoPHiAS study) (50) was carried out on a sample representative of the region; however, the included analysis of OHRQoL (50) was narrowed to elderly individuals. In the majority of the studies, adult participants were enrolled without additional age restrictions. However, two samples excluded older individuals (47, 53), while one study specifically included only participants aged 65 and over (50). In over half of the studies, the presence of the majority of teeth was required (45, 47, 48, 51, 52). The selection criteria for the studies were quite restrictive with respect to comorbidities and health risk factors: only systemically healthy individuals were qualified for six studies (46–49, 51, 53), and two studies also excluded tobacco smokers (46, 53). Periodontitis was primarily diagnosed using the 2017 consensus criteria (4, 5), except in one study (51) using the Centers for Disease Control and Prevention's older case definition (56, 57), and two with investigator-defined terms (45, 49). In one study (46), only Stage I periodontitis patients were included, whereas another (48) encompassed exclusively Stages II-IV. Localized periodontitis cases were excluded in two studies (48, 49). In one study (49), only the chronic periodontitis group was considered to prevent an overrepresentation of aggressive periodontitis in our review. Control groups in five studies included individuals with healthy periodontium (47, 48, 52, 53), in two studies comprised exclusively of individuals diagnosed with gingivitis (45, 49), and in two studies were mixed (50, 51). In one study (46), three distinct patient groups without periodontitis were included (clinical periodontal health with intact periodontium, clinical periodontal health with reduced periodontium, and gingivitis), which were combined in the primary meta-analysis scenario.

#### 3.1.4 Analyzed population

The total analyzed population included 2,287 individuals, with 1,384 periodontitis patients and 903 controls. The studies incorporated were diverse in terms of demographic and clinical characteristics of the samples analyzed (Table 2). The mean age reported in eight studies ranged from 31.6 years (49) to 72.6 years (50), and the percentage of women reported in eight studies ranged from 44.1% (52) to 75.0% (53). The proportion of smokers ranged from 0% to 37.7%; however, three studies failed to report this characteristic. The average PPD across entire study samples ranged from 0.9 mm (47) to a maximum of 4.2 mm (53), while CAL, reported in 6 studies, varied from 2.5 mm (48) to 3.8 mm (47).

**Table 2:** Main characteristics of study populations and samples.

Study	General criteria for eligibility		Exclusion criteria pertaining to:				Periodontitis group				Control group			Sample characteristics					
	Age (years)	No of remaining teeth	Smoking	Systemic diseases	Dental conditions/appliances	Previous periodontal treatment	Periodontitis definition^c^	N	Mild (stage I) periodontitis	Localized periodontitis	Type of controls	N	Gingivitis	Age (years), mean (SD)	Females	Smokers	Frequency of brushing ≤1/day	PPD (mm), mean (SD)	CAL (mm), mean (SD)
Al Habashneh et al. (45)	≥18	≥15	No	No	Yes	No	Other	233	33.9%	NR	G	167	100%	36.7 (11.9)	59.0%	23.8%	54.0%	2.4 (0.8)	2.9 (1.3)
Botelho et al. (50)	≥65	–	No	No	No	No	AAP/EFP 2017	420	20.0%	33.8%	NP	172	22.6%^d^	72.6 (6.4)	54.1%	NR	37.0%	1.9 (0.8)	3 (1.5)
Cataldo et al. (53)	35–70	–	Yes	Yes	Yes	Yes	AAP/EFP 2017	50	NR	NR	HP	50	0%	51.3 (22.2)	75.0%	0%	NR	4.2 (1.4)	NR
Dikilitaş et al. (46)	≥18	–	Yes	Yes	No	No	AAP/EFP 2017	36	100%	NR	NP^e^	130	33.8%	36.7 (7.5)	NR	0%	NR	2.8 (0.8)	3.8 (1.6)
Eroğlu et al. (47)	18–65	≥20^a^	No	Yes	Yes	Yes	AAP/EFP 2017	93	14.0%	NR	HP	31	0%	NR	58.1%	NR	46.0%	0.9 (2.4)	3.8 (0.5)
Fuller et al. (51)	–	≥20	No	Yes	No	No	AAP 2007&2012	333	27.9%	NR	NP	138	NR	38.5 (10.8)	58.0%	17.8%	NR	2.9 (1.2)	NR
Mishra et al. (48)	–	≥20	No	Yes	No	Yes	AAP/EFP 2017	50	0%	0%	HP	50	0%	41.9 (3.6)	48.0%	28.0%	55.0%	3.4 (1.6)	2.5 (2.6)
Santonocito et al. (52)	≥18	≥20	No	No	Yes	No	AAP/EFP 2017	55	NR	NR	HP	56	0%	51.8 (3.4)	44.1%	NR	NR	3.0 (1.4)	NR
Ustaoğlu et al. (49)^b^	–	–	No	Yes	No	No	Other	114	NR	0%	G	109	100%	31.6 (11.8)	53.4%	37.7%	NR	3.6 (1.1)	3.7 (1.3)

AAP, American Academy of Periodontology; CDC, Centers for Disease Control and Prevention; EFP, European Federation of Periodontology; G, gingivitis, HP, healthy periodontum, NP, non-periodontitis (G and HP mixed); NR, not reported.

^a^ Excluding third molars and retained roots.

^b^ The study comprised both “chronic” and “aggressive” periodontitis groups; only the former was included in the meta-analysis.

^c^ AAP/EFP 2017—consensus definition of the 2017 World Workshop on the Classification of Periodontal and Peri-Implant Diseases and Conditions (4, 5); CDC-AAP 2007 & 2012—definition of the CDC and AAP, proposed in 2007 and updated in 2012 (56, 57).

^d^ Approximate value, estimated based on the characteristics of a broader age group from the SoPHiAS study, reported in another publication (55).

^e^ In the Dikilitas 2023 study (46), there were three control groups with patients without periodontitis, which were pooled in the basic meta-analysis scenario.

Due to incomplete reporting, the possibility of comparing exposed groups to control groups regarding basic characteristics, including potential confounding factors, was limited (Table 3). The sample sizes of groups with periodontitis ranged from 36 (46) to 420 (50), while the control groups ranged from 31 (47) to 172 (50). In two (49, 53) out of five studies with available data, the mean age of patients with periodontitis considerably exceeded that of the non-periodontitis group by over 10 years. Among six studies that allowed for sex proportion comparison, two (47, 50) reported a notably lower percentage of women in the periodontitis groups, with one study (53) showing a substantial female predominance. As expected, the average PPD and CAL were higher in the periodontitis groups compared to control groups. These differences ranged from 1.34 mm (52) to 2.95 mm (48) for PPD and 1.80 mm (46) to 4.96 mm (48) for CAL, with data available for five and three studies, respectively.

**Table 3:** Main characteristics and severity of impacts (the mean OHIP-14 total score), reported in periodontitis (P) and control (C) groups of the included studies.

Study/Group	N		Age (years), mean (SD)		Females, *n* (%)		PPD (mm), mean (SD)		CAL (mm), mean (SD)		OHIP-14 total score, mean (SD)	
	P	C	P	C	P	C	P	C	P	C	P	C
Al Habashneh et al. (45)	233	167	NR	NR	NR	NR	NR	NR	NR	NR	12.11 (7.57)	9.5 (7.12)
Botelho et al. (50)	420	172	NR	NR	216 (51.4)	104 (60.5)	NR	NR	NR	NR	8.11 (10.6)	7.2 (10.35)
Cataldo et al. (53)	50	50	55.1 (11.1)	43.5 (12.3)	41 (82)	34 (68)	5.46 (0.78)	2.85 (0.23)	5.92 (0.42)	NR	19.1 (11.2)	7.8 (7.3)
Dikilitaş et al. (46)	36	130	39.5 (5.17)	35.91 (7.9)	NR	NR	4.19 (0.15)	2.46 (0.31)	5.17 (0.21)	3.37 (1.56)	13.03 (3.47)	5.3 (5.24)
Eroğlu et al. (47)	93	31	NR	NR	50 (53.8)	22 (71)	NR	NR	NR	NR	11.39 (13.15)	7.7 (9.01)
Fuller et al. (51)	333	138	NR	NR	NR	NR	NR	NR	NR	NR	13.23 (10.32)	5.2 (6.62)
Mishra et al. (48)	50	50	43.61 (3.28)	40.24 (3.04)	25 (50)	23 (46)	4.91 (0.57)	1.96 (0.45)	4.98 (1.26)	0.02 (0.01)	17.02 (9.99)	6.3 (5.59)
Santonocito et al. (52)	55	56	52.1 (3.6)	51.5 (3.2)	23 (41.8)	26 (46.4)	3.65 (1.4)	2.31 (1.1)	NR	NR	11.89 (2.5)	6.9 (2.1)
Ustaoğlu et al. (49)	114	109	39.23 (11.32)	23.71 (5.27)	63 (55.3)	56 (51.4)	4.56 (0.6)	2.57 (0.3)	4.89 (0.5)	2.48 (0.3)	13.53 (9.38)	7.1 (5.03)

C, control group; CAL, clinical attachment loss; NR, not reported; P, periodontitis group; PPD, probing pocket depth; SD, standard deviation.

#### 3.1.5 Study quality

The quality of all studies was assessed as “Fair” (Supplementary Table S5, Figure S1). The most common limitations included: the inability to determine the duration of exposure (i.e., the duration of periodontitis), determining whether the diagnosis preceded the OHRQoL assessment, and whether outcome assessors were blinded to the exposure status of the participants. In more than half of the studies, the relationship of OHRQoL to different levels of exposure (i.e., stages of periodontitis progression) was not assessed, and in most cases, confounding variables were not measured and/or reported in sufficient detail.

#### 3.1.6 OHRQoL data validity

In the included studies, country-specific language versions of the OHIP-14 were utilized. Seven studies (46–52) employed previously validated OHIP-14 versions in different populations. In one instance, a new language version of the OHIP-14 was adapted and validated for the study (45). In another study (49), explicit information on prior validation was not provided; however, the internal consistency was assessed and found to be adequate. Overall, four included studies (45, 47–49) evaluated the internal consistency of all OHIP-14 items in the study samples, yielding satisfactory results in each case (Cronbach's alpha ≥0.70).

#### 3.1.7 OHRQoL results

Each study included in the review assessed severity of impact (OHIP-14 total score), while impact prevalence of domains was evaluated only in two (45, 51). Extent of domains was not examined in any of the reviewed studies. The mean OHIP-14 total score ranged from 8.1 (50) to 19.1 (53) in the periodontitis groups and from 5.2 (51) to 9.5 (45) in the control groups. Across studies, it consistently showed higher scores in exposed groups compared to non-exposed groups. However, there was noticeable variability in the size of these differences, ranging from as small as 0.96 (50) to a maximum difference of 11.3 (53). The detailed results from individual studies can be found in Table 3 and Supplementary Table S6.

#### 3.1.8 Reporting bias

The funnel plot showed a wide spread of results, but no noticeable asymmetry (Supplementary Figure S2). Egger's test for publication bias indicated no significant bias (bias coefficient = 2.28, 95% CI: −2.95 to 7.50, *P* = 0.337).

### 3.2 Results of the meta-analyses

#### 3.2.1 Overall effect

**Figure 2:**
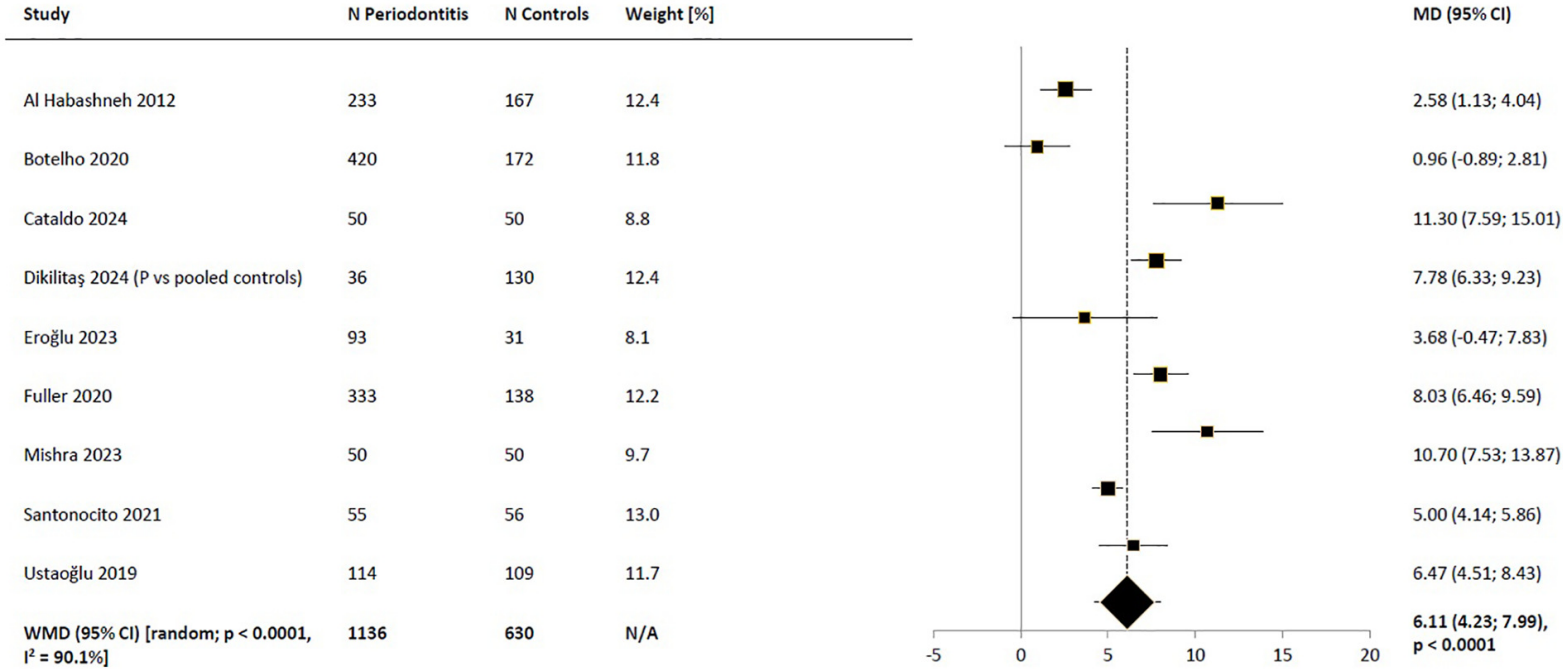
Weighted mean difference in severity of impacts (mean OHIP-14 total score) in periodontitis patients compared to control subjects, meta-analysis of the results of all included studies. WMD, weighted mean difference; MD, mean difference; CI, confidence interval; N/A, not applicable; P, periodontitis.

The combined results of all nine studies included in the systematic review indicated that periodontitis had a significant impact on the mean OHIP-14 total score when compared to controls with healthy periodontium or gingivitis. The OHIP-14 total score was over 6 points higher in patients with periodontitis [WMD_random_ = 6.11 (95% CI: 4.23; 7.99), *p* < 0.0001; Figure 2]. However, there was high heterogeneity among studies (*I*^2^ = 90.1%, *p* < 0.0001). In the *post-hoc* sensitivity analysis, excluding any single study did not result in a reduction of heterogeneity (data not shown).

#### 3.2.2 Results in subgroups by region, definition and stages of periodontitis, and type of controls

**Figure 3:**
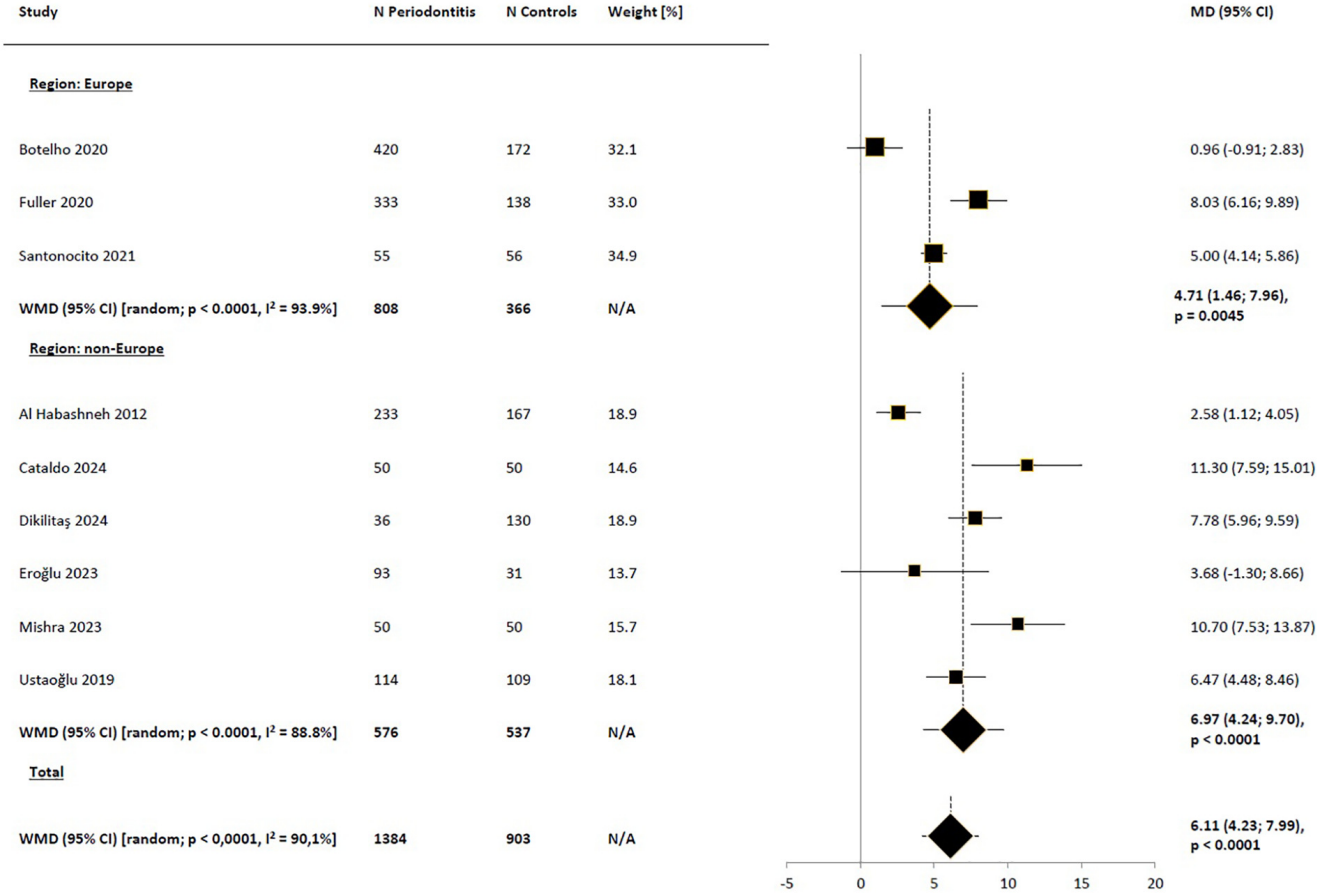
Weighted mean difference in severity of impacts (mean OHIP-14 total score) in periodontitis patients compared to control subjects, meta-analyses in subgroups of studies by region. WMD, weighted mean difference; MD, mean difference; CI, confidence interval; N/A, not applicable.

**Figure 4:**
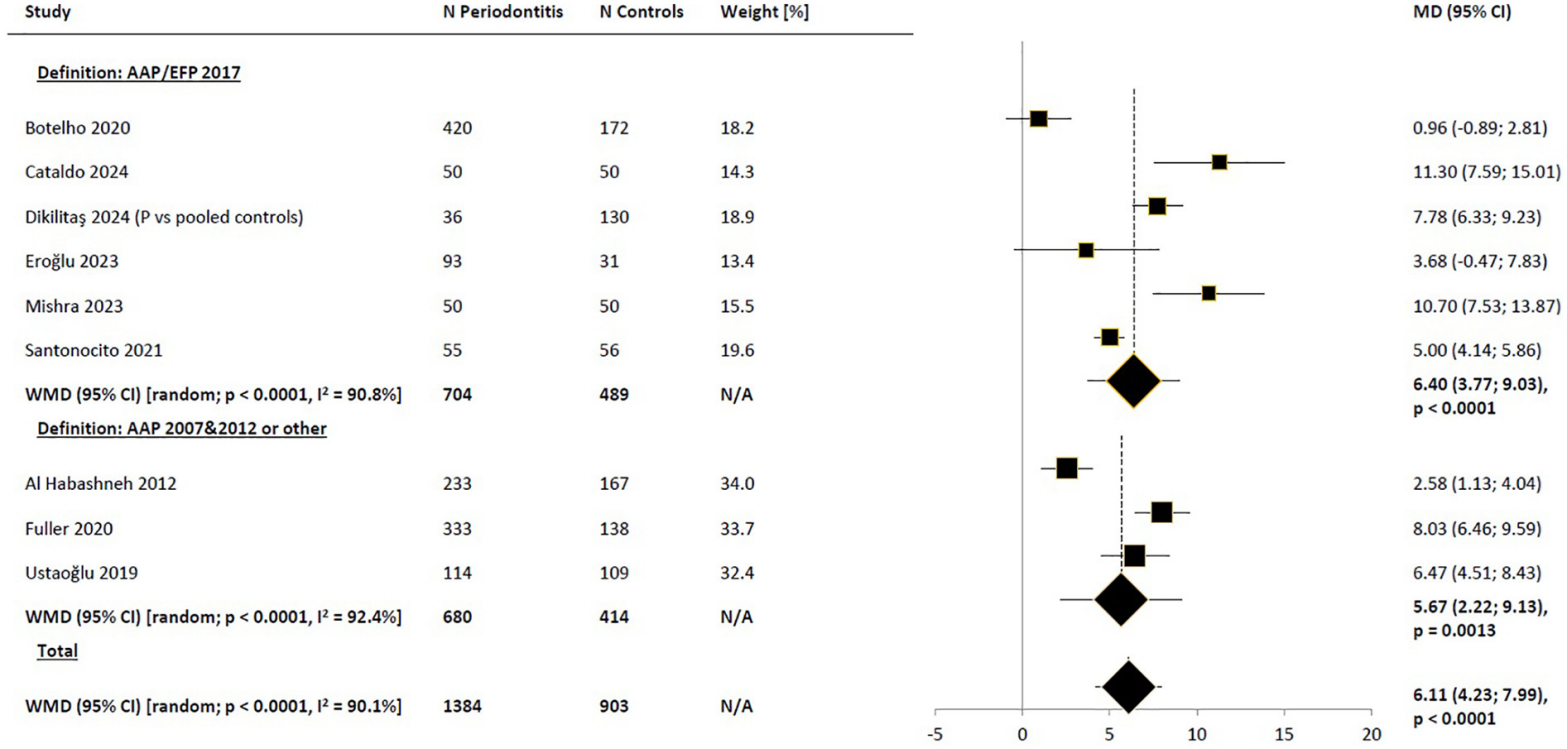
Weighted mean difference in severity of impacts (mean OHIP-14 total score) in periodontitis patients compared to control subjects, meta-analyses in subgroups of studies by adopted definition of periodontitis. WMD, weighted mean difference; MD, mean difference; CI, confidence interval; N/A, not applicable; P, periodontitis; AAP, American Academy of Periodontology; EFP, European Federation of Periodontology.

**Figure 5:**
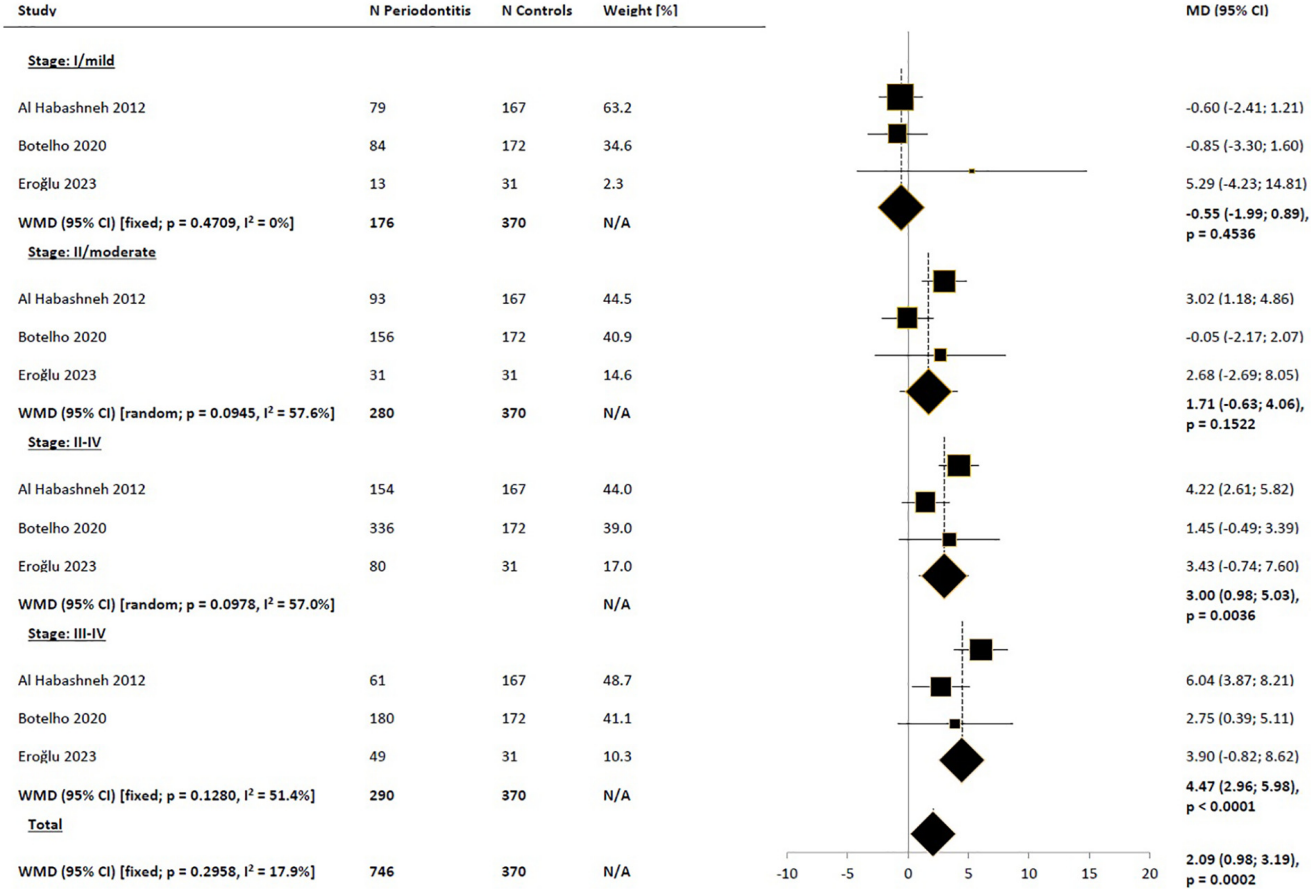
Weighted mean difference in severity of impacts (mean OHIP-14 total score) in periodontitis patients compared to control subjects, meta-analyses in subgroups by the periodontitis stage; only studies with available results for subgroups were included. WMD, weighted mean difference; MD, mean difference; CI, confidence interval; N/A, not applicable.

**Figure 6:**
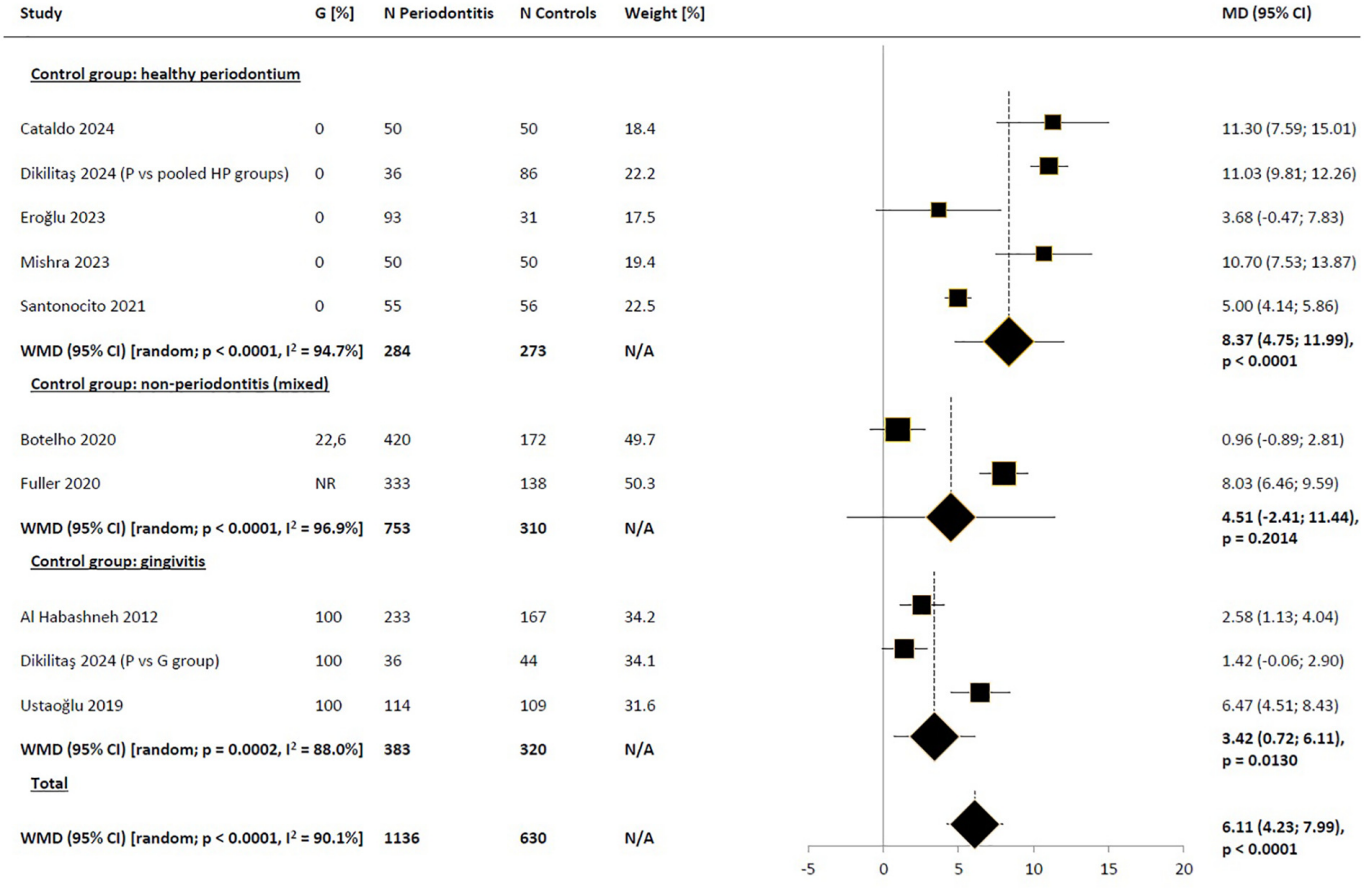
Weighted mean difference in severity of impacts (mean OHIP-14 total score) in periodontitis patients compared to control subjects, meta-analyses in subgroups of studies by the type of control group with respect to the inclusion of gingivitis patients. WMD, weighted mean difference; MD, mean difference; CI, confidence interval; N/A, not applicable; P, periodontitis; G, gingivitis; HP, healthy periodontum.

Subgroup analysis based on regions (Europe vs. non-Europe) did not suggest that the observed differences in results were due to regional variations (Figure 3). Narrowing the pool of studies to those where the diagnosis of periodontitis was based on the American Academy of Periodontology/European Federation of Periodontology (AAP/EFP) consensus definition from 2017 also did not reduce heterogeneity, yielding very similar difference to those obtained in the base-case scenario [WMD_random_ = 6.40 (95% CI: 3.77; 9.03), *p* < 0.0001; Figure 4]. Comparison of the impact of periodontitis on OHRQoL according to disease stage was feasible based on a small subset of 3 studies (45, 47, 50). Pooled results suggested a trend toward higher differences in OHIP-14 total scores between the periodontitis groups in advanced stages, compared to the control groups, as expected, with no significant impact on OHRQoL observed in stage I (Figure 5). Notably, this trend persisted even though the pooled effect size calculated from the overall samples of the 3 studies reporting results by disease stages indicated a noticeably lower impact of periodontitis on OHRQoL compared to the main meta-analysis, which included all eligible studies (WMD = 2.09 and 6.11, respectively). The selection of individuals for the control group also appeared to influence the investigated relationship. The pooled difference in total OHIP-14 scores was the greatest in studies where the control group consisted of patients with a healthy periodontium, the smallest when comparing periodontitis patients to groups with gingivitis, and intermediate in comparisons vs. mixed groups (WMD = 8.37, 3.42, and 4.51, respectively). However, the results within the analyzed subgroups remained highly heterogeneous (Figure 6).

#### 3.2.3 Subgroups and meta-regression analyses by age

Meta-analyses conducted within subgroups of studies based on the age difference between the periodontitis group and the control group (data available for 5 studies) did not indicate a relationship between the observed age difference and the mean difference in total OHIP-14 scores (Supplementary Figure S3). Furthermore, in the univariate meta-regression analysis, the age difference between groups did not act as a significant modifier of OHIP-14 differences (Supplementary Table S7, Figure S4). Conversely, the difference in mean total OHIP-14 score tended to decrease with increasing mean age of the total sample (nonsignificant numerical trend, 8 studies; Supplementary Table S7, Figure S5). This trend was, however, mitigated in the bivariate model, incorporating both the mean age of the entire sample and the age difference (5 studies; Supplementary Table S7).

#### 3.2.4 Subgroups and meta-regression analyses by sex

Meta-analyses conducted within subgroups of studies stratified by the direction of between-group differences in the percentage of women (6 studies) suggested that sex might modify the impact of periodontitis on the assessed measure of OHRQoL (Supplementary Figure S6). The weighted mean difference in mean total OHIP-14 score in the subgroup of studies where the periodontitis group had a higher percentage of women than the control group was 8.26 (fixed model; 95% CI: 6.47, 9.78; *p* < 0.0001). In studies with a higher percentage of women in the control group, the corresponding WMD value was 3.23 (random model, 95% CI: 0.15, 6.31; *p* = 0.0396). Importantly, the impact of periodontitis on OHRQoL remained statistically significant in both analyzed subgroups. In univariate meta-regression analyses, the intergroup difference in the percentage of women (6 studies) served as a statistically significant predictor of greater OHIP-14 difference (the regression coefficient = 0.337; *p* < 0.001; Supplementary Table S7, Figure S7), while such a relationship was not observed for the mean percentage of females across the entire sample (8 studies; Supplementary Table S7, Figure S8). In a bivariate meta-regression model that included both discussed variables (6 studies), the difference in the proportion of females between groups retained its status as a significant predictor of total OHIP-14 score difference (the regression coefficient = 0.335; *p* = 0.004), independently of the mean percentage of females across the entire sample (Supplementary Table S7).

#### 3.2.5 Subgroups and meta-regression analyses by probing pocket depth

As expected, a larger between-group difference in mean PPD was associated with an increased observed impact of periodontitis on mean total OHIP-14. This was evident both in subgroup analyses based on the difference in PPD between the periodontitis group and the control group (data available from 5 studies, Supplementary Figure S9), as well as in univariate (5 studies; regression coefficient = 3.741; *p* < 0.001; Supplementary Table S7, Figure S10) and bivariate (5 studies; regression coefficient = 4.426; *p* = 0.002; Supplementary Table S7) meta-regression analyses. The mean PPD value in the sample was a significant predictor of OHRQoL only in the single-factor model (9 studies; the regression coefficient = 3.088; *p* = 0.001; Supplementary Table S7, Figure S11); after also considering the difference in PPD in the two-factor model, only the second of the mentioned variables retained its status as a significant predictor.

#### 3.2.6 Feasibility constraints and limitations

Considering the limited number of studies that could be included in the models (especially for sex and age), the results of the presented meta-regression analyses should be treated as exploratory. Due to insufficient availability of sample and group characteristics, conducting a meta-regression analysis with a greater number of potential predictors was not feasible.

Due to the limited prevalence data reported by only two studies, a meta-analysis for the intended dichotomized outcome could not be performed.

## 4 Discussion

In our meta-analysis, we included rigorously selected studies based on predefined criteria, resulting in a pool of the best available comparative studies assessing the impact of periodontitis, diagnosed according to contemporary criteria, on OHRQoL measured with the OHIP-14 questionnaire. The pooled result of all included studies indicated that OHRQoL was worse in individuals with periodontitis compared to those without this condition. Assessing the clinical significance of this result can be challenging due to the lack of a universally applied method for determining the minimal clinically important difference or minimal important difference (MCID/MID) (58). In the spectrum of studied populations (including generally healthy students, prosthodontic and orthodontic patients, removable partial denture wearers, and patients with Behçet's disease) and approaches to determine MCID (various anchor-based and distribution-based methods), reported MCID/MID for OHIP-14 varied widely from 1.68 (59) to 15 points (60). However, the upper limit of this range seemed to be an outlier; the other identified values fell within the range of 1.68–4.45 points (59, 61–66). Conservatively adopting an MCID value of 4.45 points, the unadjusted difference between mean total OHIP-14 scores in the periodontitis group compared to the control group (WMD = 6.1 points) should be considered clinically significant. In subgroup analysis based on periodontitis stage, MCID threshold was not exceeded in subgroups with stages I and II, although data for subgroups were available only from 3 studies; additionally, the pooled result for stage I periodontitis significantly deviated (in the negative direction) from the main scenario. Based on our findings, we could not definitively conclude whether only stage III periodontitis led to clinically significant reduction in OHRQoL.

Our main pooled estimate of the raw difference in total OHIP-14 scores between periodontitis patients and non-periodontitis controls indicated a slightly stronger deterioration in OHRQoL compared to the result obtained from the meta-analysis by Paśnik-Chwalik and Konopka (31) which reported a difference of 4.2 points (95% CI: 3.10; 5.31). The cited result derived from pooling data from 7 studies, of which only 3 overlapped with our dataset. The relative consistency of our findings with the older meta-analysis suggested that including studies based on more contemporary definitions and stricter criteria for clinical examination did not alter the conclusion regarding the impact of periodontitis on significant OHRQoL impairment. On the contrary, the observed association in newer studies was even more evident. Furthermore, in our study pool, similar to the work of Paśnik-Chwalik and Konopka (31), worsening OHRQoL was associated with increasing severity of periodontitis. Due to the use of different statistics (WMD vs. pooled OR, respectively), direct comparison of pooled estimates for subgroups was not feasible. However, achieving consistent conclusions across different datasets and using distinct indicators remained a strong indication of the robustness of the obtained results, regardless of methodological assumptions in the review.

Since random allocation of patients to groups with and without periodontitis was clearly impossible, we obtained an estimator for the deterioration of OHRQoL due to periodontitis by pooling results from non-randomized studies. Most of these studies compared periodontitis groups to non-matched controls. Thus, it was essential to consider whether the unadjusted difference in total OHIP-14 scores truly reflected the pure effect of periodontitis or if it was confounded by other differences between exposed and non-exposed groups. Cochrane Collaboration recommended selecting estimates adjusted for confounders in meta-analyses of non-randomized studies, if available (67, 68). However, other authors considered it inappropriate to combine odds ratios adjusted to different sets of covariates (69). In a previous meta-analysis (31), the authors attempted to address potential confounding by pooling adjusted estimators obtained from regression analyses in primary studies. Specifically, they pooled adjusted odds ratios (aORs) from five primary studies, resulting in a statistically significant cumulative aOR of 1.33 (31). Although the dependent variable for these calculations was not explicitly defined in the cited work, it was reasonable to assume that it was the prevalence of domains (i.e., the percentage of study participants reporting “fairly often” or “very often” on one or more items of the OHIP-14 questionnaire). The mentioned result indicated the persistence of an association between the presence of periodontitis and OHRQoL, even under conditions of at least partial control for confounding variables. In our review, we applied more rigorous inclusion criteria for studies, resulting in the inclusion of only one study (51) out of five from which adjusted odds ratios (aOR) were extracted for the meta-analysis by Paśnik-Chwalik and Konopka (31). Among the 9 eligible for inclusion, only two (45, 51) reported the prevalence of domains divided into groups with and without periodontitis. Overall, during systematic selection, a total of 6 studies were identified that reported the relationship between periodontitis diagnosis and assessment of OHRQoL using the OHIP-14 questionnaire assessed through multifactorial regression analysis while simultaneously meeting all other inclusion criteria. In two of these studies (53, 70), the OHIP-14 score was not a dependent variable (“outcome”) but a potential predictor of periodontitis. In the next two studies (52, 63), the outcome was a continuous variable defined as the OHIP-14 total score. In one study, the dependent variable was dichotomized based on median OHIP-14 score (71), and in the last study, regression analysis results were shown exclusively for periodontitis vs. healthy groups based on the outdated 1999 definition (51). Therefore, meta-analyzing adjusted estimators from primary studies was not possible, and three studies that did not contain other data suitable for inclusion in the meta-analysis (70–72), were excluded.

We attempted to assess the impact of confounding variables on the results of the meta-analysis using an alternative approach, through subgroup analyses and meta-regression. What might seem unexpected, subgroup analysis and meta-regression results did not definitively demonstrate an impact of age differences between patients with periodontitis and control groups on the assessed OHRQoL parameter. In the only study with age-matched controls (52), the mean difference in the mean total OHIP-14 score was numerically smaller than in the other studies with available data, although it remained statistically significant. This suggested that the observed deterioration in OHRQoL among patients with periodontitis was not a result of the confounding effect of higher average age in the exposed groups. The difference in mean total OHIP-14 score tended to decrease with increasing mean age of the entire sample, although this effect was mitigated in the bivariate model that considered both the mean age of the entire sample and the age difference. However, the impact of age might have been obscured due to the inclusion of a smaller number of studies in the analysis. In line with our results, available literature did not indicate a clear association between older age and a decline in OHRQoL (73–75). On the contrary, several studies have reported better OHRQoL among older individuals compared to younger or middle-aged people (36, 76–78). Interestingly, as shown in a meta-analysis based on a systematic review (40), this effect was also observed in age subgroups within the older adult population. Attempts to explain the lack of negative impact of age on OHRQoL focused on mediation through personal and environmental factors, adaptive processes, and coping mechanisms (40, 79).

In our study, sex differences appeared to be relevant for assessing the impact of periodontitis on OHRQoL. A higher percentage of women in the periodontitis group was associated with greater deterioration in OHIP-14 scores compared to the control group. Previous studies showed instead that male sex was a risk factor for both developing periodontitis and experiencing more advanced disease stages (6, 80). Women tended to exhibit better oral hygiene practices and generally had improved oral health (80). However, these associations did not necessarily translate into a more positive perception of OHRQoL among women. To the best of our knowledge, the impact of sex on OHRQoL remained unresolved at this time (81). Several previous studies reported that women might have worse OHRQoL than men (82, 83). On the contrary, men more frequently reported poor oral health compared to women (84), or no statistically significant differences were found between the sexes, although numerical trends suggested poorer self-assessment results among women (85, 86). The results of our meta-analysis suggested that female sex might act as a factor associated with worse OHRQoL.

The results of measuring the association between PPD and the impact of periodontitis on OHRQoL were consistent with expectations. A greater difference in PPD between the exposed group and the control group was associated with a larger intergroup difference in total OHIP-14 score, favoring the non-periodontitis group. This effect was seen in both subgroup analyses and meta-regression analyses. Considering the changes that occurred over the years in the criteria for assessment of periodontal status (4, 5), it would be interesting to compare the significance of PPD to other clinical characteristics, particularly CAL. Unfortunately, poor reporting in the included studies precluded a comprehensive analysis of this kind.

In our meta-analysis, significant heterogeneity was evident, as indicated by an I^2^ value close to or exceeding 90% in the main scenario and most analyzed subgroups. No studies were clearly identified as outliers that, if excluded from the meta-analysis, would lead to a substantial reduction in heterogeneity. Overall, the observed variability could not be fully explained by regional differences, study designs, inclusion criteria, case definitions of periodontitis (whether based on the 2017 consensus or other criteria), or sample characteristics. Notably, subgroups based on periodontitis stages were an exception. However, it was uncertain whether this was a side effect of significantly reducing the number of studies reporting results for stages compared to the main scenario. Additionally, apart from potential variations in the proportion of exposed patients across different stages of periodontitis, subgroup analysis also highlighted the importance of diverse participant selection criteria for control groups. As mentioned earlier, the pooled difference in total OHIP-14 scores was the greatest in studies where the control group consisted of patients with a healthy periodontium, the smallest when comparing periodontitis patients to groups with gingivitis, and intermediate in comparisons vs. mixed groups. This suggested that the presence of gingivitis was associated with a certain deterioration in OHRQoL detectable using OHIP-14. In a systematic review without meta-analysis, Ferreira et al. showed evidence indicating the impact of gingivitis on OHRQoL, although weaker than that for periodontitis (32). Our results were consistent with this conclusion.

The important assumption of our review and meta-analysis methodology was to operationalize the latent variable represented by OHRQoL through indicators calculated based on patient responses to OHIP-14 questionnaire. This questionnaire was chosen due its documented reliability and validity (87–90), as well as its frequent use in studies assessing the impact of periodontal diseases on quality of life, including OHRQoL (15). Additionally, the potential availability of various OHIP-14-derived indices (i.e., severity, prevalence, and extent of domains) could allow for a more comprehensive description of the influence of periodontitis on OHRQoL. However, ultimately, only the severity of domains (total OHIP score) was consistently measured and uniformly reported in the available studies. We decided against pooling results from OHRQoL assessments conducted using different tools, including alternative versions of OHIP-14, the Oral Impacts on Daily Performance (ODIP) scale, or the Geriatric Oral Health Assessment Index (GOHAI). Allowing diverse measurement instruments would introduce further heterogeneity, and the practical applicability of standardized pooled estimates appear challenging.

The strengths of our synthesis lie in conducting a meta-analysis with a substantial inclusion of new studies, where periodontitis was diagnosed based on the current consensus criteria from 2017 (4, 5). We thoroughly selected studies based on the method of assessing periodontal status. Unlike previous systematic reviews (34–37), we exclusively considered studies with a reliable clinical diagnosis, involving at least 4 measurement points per tooth and full-mouth evaluation. As it had been found, partial-mouth recording protocols, while allowing time and resource savings, might lead to biased estimates of periodontal disease prevalence and severity (91). We excluded studies that assessed periodontal status using indices such as the CPI, as relying on the worst sextant in such assessments could result in overdiagnosis of periodontitis and, consequently, a false conclusion regarding its association with impaired OHRQoL (32, 92). Furthermore, due to low specificity and the risk of missing the detection of mild to moderate periodontitis (5), we did not include studies in which patient classification into the periodontitis group relied solely on radiographic assessment (38) or with the simultaneous presence of caries in all patients with periodontitis (39). Despite certain limitations stemming from the suboptimal quality of reporting in primary studies, we systematically and quantitatively evaluated the significance of periodontal stages, patient selection for the control group, and key demographic characteristics of participants (age and sex) in relation to the investigated association between periodontitis and OHRQoL.

The results of our meta-analysis confirmed that periodontitis significantly impaired OHRQoL with potential association with disease severity and female sex. As aging of the world's population, especially in high-income countries, is expected in the forthcoming decades, the significance of periodontitis and its impact on quality of life will likely become more important for public health than nowadays (93). Besides periodontitis does not pose an immediate risk of mortality, it is certainly responsible for prolonged suffering and pain, resulting in both psychological and aesthetic problems (94). Expanding knowledge about periodontitis among health care professionals as well as their patients seems crucial as accurate treatment has been shown to improve cardiovascular risk and reduce systemic inflammation (95). Therefore, our meta-analysis could be perceived as a valuable addition to help in developing national strategies for combating diseases of public health relevance that could be of significant benefit for both patients and health costs worldwide (8).

Several limitations related to the measurement properties of the OHIP-14 warrant consideration when interpreting the findings of this systematic review. These limitations pertain to the nature of the construct being measured, the core principles of psychometric assessment, and recent evidence regarding the instrument's performance across diverse populations. OHRQoL, as a latent construct, is not directly observable and must be inferred from responses to scale items. Consequently, evidence of validity and reliability from one sample or context does not automatically ensure the same level of measurement quality when the instrument is applied to another sample with differing demographic characteristics, cultural backgrounds, or clinical conditions. While the OHIP-14 is a widely used instrument with foundational validation studies often cited, a growing body of literature highlights considerable variability in its psychometric performance across diverse samples and settings (20, 96, 97). For example, there is evidence indicating that the OHIP-14 may function differently in dental patient samples compared to non-dental patient or general population samples, with superior performance frequently observed in clinical groups seeking care (97). Several studies have questioned the fit of the original seven-factor structure proposed during the scale's development (22). Evidence occasionally supports alternative models, such as unifactorial structures, where all items align with a single general OHRQoL factor, as well as three- or four-factor configurations, with the strongest evidence currently favoring the four-dimensional structure (22). Cross-cultural and linguistic applications have also revealed variability, indicating that cultural context can influence how items are interpreted and how the underlying structure manifests (98). Overall, the available data suggest that the instrument is sensitive to the specific characteristics of the sample and the context in which it is applied. As a result, it may not function as a universally equivalent measure of OHRQoL across all the diverse populations typically included in systematic reviews, potentially measuring slightly different aspects of the construct or measuring the same construct with varying degrees of precision in different studies. This does not necessarily invalidate the OHIP-14 but rather highlights its context-dependent nature. Apparent variability in the psychometric properties of the OHIP-14 across different studies represents a potential limitation for this systematic review, especially in relation to the synthesis and comparison of findings. The core assumption when pooling results is that the outcome measure assesses the same underlying construct in the same way across all included studies. If the OHIP-14 did not measure the OHRQoL construct equivalently across the primary studies included, the meta-analysis results could be affected by measurement bias. Such measurement non-equivalence implies that observed differences in scores between groups may not exclusively represent true disparities in OHRQoL, but could instead be partially or entirely due to the measurement instrument functioning differently in periodontitis patients compared to healthy controls. This phenomenon can act as a significant, unmeasured source of heterogeneity, potentially inflating statistical heterogeneity measures and making it difficult to discern whether observed variability in outcomes is due to true clinical or methodological differences between studies, or simply measurement artifacts. While the reporting standards in primary studies often precluded a formal, study-by-study assessment of the validity and reliability of the OHIP-14 data using established criteria [e.g., the COSMIN Risk of Bias checklist and criteria for good measurement properties (99)], awareness of the potential for measurement variability informed the qualitative synthesis of the findings. Variations in reported OHIP-14 scores were interpreted with caution, acknowledging that differences could stem from measurement issues as well as substantive factors. Statistical heterogeneity in our meta-analyses was substantial. While subgroup analyses and meta-regression indicated that variations in disease stage, characteristics of the control groups, sex, and PPD might partially explain the observed inconsistency in results, significant residual heterogeneity persisted. It is plausible that underlying psychometric variability in the OHIP-14 across studies contributed to this heterogeneity. Addressing the issue of measurement equivalence rigorously would ideally involve testing for measurement invariance across studies prior to data pooling, using techniques such as multi-group confirmatory factor analysis (100, 101). However, conducting such analyses retrospectively within a systematic review context presents significant practical challenges. It typically requires access to individual participant data or, at minimum, detailed psychometric information such as item-level covariance matrices and means or thresholds from each primary study included in the review. Such granular data are rarely reported in published articles or made available by primary study authors. Furthermore, adequate sample sizes within each primary study are needed for stable estimation, and the quality of reporting on the instrument's use is often insufficient. Consequently, formal cross-study measurement invariance testing was not feasible for this review due to the unavailability of the necessary data. It is also worth noting that while measurement invariance is a critical theoretical concept, there are ongoing discussions within the psychometric literature regarding the practical implications of minor violations of invariance and the potential limitations of traditional measurement invariance testing approaches, including their sensitivity to large sample sizes and the interpretation of partial invariance (102, 103). Nevertheless, the inability to formally assess measurement invariance across the included studies represents a limitation in establishing the strict measurement equivalence of the OHIP-14 data being synthesized. The potential impact of measurement non-equivalence should therefore be considered when interpreting the pooled results and overall conclusions of this review.

Our review has certain further limitations, most of which originate from the practical constraints inherent in available research. We assessed the quality of all studies included in the review as “fair”. Only one out of the nine included studies had a matched control group, but its quality was also rated as “fair” due to other shortcomings. Some of the included studies attempted to mitigate the impact of potential confounders using statistical methods, but the diversity of applied methods precluded the use of reported adjusted estimates in our meta-analyses. The feasibility of employing an alternative valid approach to address the influence of confounding variables, such as subgroup analyses and meta-regression, was limited by the scarcity of data. We were unable to assess the significance of several potentially relevant factors, such as socioeconomic status, education level, tobacco use, presence of comorbidities, or adherence to oral hygiene practices. Among the standard clinical parameters characterizing periodontal status consistently reported in primary studies, only PPD was available for both groups with and without periodontitis, making it impossible to estimate the impact of differences in CAL, bleeding on probing or plaque index on OHRQoL detriment. Given our goal of calculating pooled estimators, several studies were excluded solely due to non-standard presentation of results, rendering their estimates unusable for our meta-analyses. The number of studies meeting inclusion criteria was too low to conduct multivariate meta-regression considering multiple sociodemographic and clinical factors.

There are also particular methodological constraints inherent in our work. First, our review did not have a protocol registered in advance. The absence of a pre-registered protocol may introduce potential bias, as methodological decisions could be adjusted *post hoc*. We mitigated this risk by adhering strictly to our predefined inclusion criteria and the PRISMA reporting guidelines, but transparency is nonetheless reduced without a publicly available protocol. Second, we chose not to contact study authors for additional data. While obtaining unpublished data directly from authors can make a review more complete and reduce reporting bias (104), this was not pursued due to feasibility constraints (time and resource limitations). As a result, some studies with incomplete reporting had to be excluded, which could influence our findings. This approach might bias the meta-analysis toward studies with more complete data and limit the generalizability of results. We acknowledge that these choices—lack of protocol registration and not retrieving missing data from authors—are limitations of our review, and they may impact the rigor and applicability of the conclusions*.* In our review, we included only studies with full-text publications. This criterion may have introduced bias, as it excluded studies conducted on smaller samples or those that did not yield statistically significant results. However, grey literature, such as conference abstracts and letters, was unlikely to contain sufficiently precise numerical data to be deemed eligible for our meta-analyses. Notably, even full-text articles often provided insufficient data for comprehensive analysis. We were also unable to include publications written in languages other than English and Polish, which may have introduced bias by excluding studies with negative results and reducing the representation of patients from certain geographical regions. Finally, meta-analysis of dimension-specific results could provide more insights into the impact of periodontitis across the different dimensions of OHRQoL and might be an interesting option for future research in this area (105).

## 5 Conclusions

In conclusion, our findings confirmed that periodontitis significantly and clinically worsened OHRQoL, which was an important component of general quality of life, with potential association with disease severity and female sex. However, our review also revealed a rare occurrence of original studies attempting to control for confounding variables at the study design level, such as comparing cohorts matched for known and most likely confounding factors related to the studied association. This situation hinder a reliable comprehensive assessment of the mediators of periodontitis impact on OHRQoL and call for the development and adherence to uniform standards in future research conducted in this area.

## Data Availability

Publicly available datasets were analyzed in this study. This data can be found here: We systematically searched the PubMed, Embase, and Scopus databases up to March 8, 2024.
