# Supplementary material for: Oral health-related quality of life in patients with periodontitis: a systematic review and meta-analysis
Source: Front Oral Health. 2025 May 26;6:1503829. doi: 10.3389/froh.2025.1503829 (PMC12146335; doi:10.3389/froh.2025.1503829)
Supplement: Supplementary file 1 [file Datasheet1.pdf]

Supplementary materials

Oral Health-Related Quality of Life in Patients with Periodontitis: A Systematic Review and Meta-Analysis

Table S1. PubMed search strategy 3
Table S2. Embase search strategy 3
Table S3. SCOPUS search strategy 3
Table S4. Selection criteria defined in a PECOS scheme 4
Table S5. Quality of the included studies (risk-of-bias), based on the Quality Assessment Tool for Observational Cohort and Cross-Sectional Studies developed by NHLBI (1) 6
Table S6. Detailed characteristics and outcomes of the included studies 7
Table S7. Results of the univariate and bivariate meta-regression analyses for the mean difference in total OHIP score (periodontitis versus controls), random model 17
Figure S1 . Risk-of-bias traffic plot, based on the Quality Assessment Tool for Observational Cohort and Cross-Sectional Studies developed by NHLBI (1) 19
Figure S2 . Risk of bias due to missing results (funnel plot) 20
Figure S3. Weighted mean difference in severity of impacts (mean OHIP-14 total score) in periodontitis patients compared to control subjects, meta-analyses in subgroups of studies by the difference in mean age between P and C groups; only studies with available mean age for P and C groups were included...21
Figure S4 . Prediction line determined by meta-regression for difference in mean age (periodontitis group versus control group) as an independent variable....................................22
Figure S5 . Prediction line determined by meta-regression for mean age of the sample as an independent variable...23
Figure S6 . Weighted mean difference in severity of impacts (mean OHIP-14 total score) in periodontitis patients compared to control subjects, meta-analyses in subgroups of studies by group with the higher percentage of females; only studies with available percentage of females for P and C groups were included 24
Figure S7 . Prediction line determined by meta-regression for difference in percentage of females (periodontitis group versus control group) as an independent variable....................................25
Figure S8 . Prediction line determined by meta-regression for percentage of females in the sample as an independent variable..26
Figure S9 . Weighted mean difference in severity of impacts (mean OHIP-14 total score) in periodontitis patients compared to control subjects, meta-analyses in subgroups of studies by the difference in mean PPD between P and C groups; only studies with available mean PPD for P and C groups were included...27
Figure S10 . Prediction line determined by meta-regression for difference in mean probing pocket depth (periodontitis group versus control group) as an independent variable 28
Figure S11 . Prediction line determined by meta-regression for mean probing pocket depth in the sample as an independent variable..29
Table S8. PRISMA 2020 Checklist (25)..30
References...34

Search strategies

Table S1. PubMed search strategy

#1	"oral health impact profile"[all]
#2	ohip[tw]
#3	"oral health related quality of life"[all]
#4	#1 OR #2 OR #3
#5	"Periodontal Diseases"[mh] OR "Periodontitis"[mh] OR Periodontitis[tw] OR Periodontal[tw]
#6	#4 AND #5

Table S2. Embase search strategy

#1	'oral health impact profile' AND [embase]/lim
#2	'ohip' AND [embase]/lim
#3	'oral health related quality of life' AND [embase]/lim
#4	#1 OR #2 OR #3
#5	('periodontal disease'/exp OR 'periodontitis' OR 'periodontal') AND [embase]/lim
#6	#4 AND #5

Table S3. SCOPUS search strategy

#1	TITLE-ABS-KEY ("oral health impact profile")
#2	TITLE-ABS-KEY (ohip)
#3	TITLE-ABS-KEY ("oral health related quality of life")
#4	#1 OR #2 OR #3
#5	TITLE-ABS-KEY (periodontitis OR periodontal)
#6	#4 AND #5

Table S4. Selection criteria defined in a PECOS scheme

PECOS	Inclusion criteria	Exclusion criteria
**P**opulation	The general population of adults (≥18 years old), not selected for any specific disease/condition (with the exception of periodontitis and gingivitis) or comorbidity	— Children or adolescents<br>— Patients selected according to the specific comorbidity (e.g. diabetes, rheumatoid disease, malignancy, caries) or physiological condition (pregnant women, women in post-partum period)
**E**xposure (group of exposed people)	Periodontitis, diagnosed based on a definition that includes at least probing pocket depth (PPD) and clinical attachment loss (CAL), as determined by a full-mouth clinical examination (of all teeth, excluding third molars), with at least 4 measurement points per tooth	— Lack of periodontitis group<br>— Inadequate periodontitis definition: definition of periodontitis based on the Community Periodontal Index of Treatment Needs (CPITN), the Community Periodontal Index (CPI) or Periodontal Screening and Recording / Periodontal Screening Index (PSR/PSI), based solely on CAL or PPD, based solely on bone loss (BL) in radiographs or based on an outdated definition from 1999 or before, lack of periodontitis definition<br>— Inadequate periodontal examination: partial-mouth examination, less than 4 measurement points or insufficient information, no clinical examination (self-reported periodontitis only, radiographic assessment only)<br>— Periodontitis cases mixed with gingivitis cases ("periodontal diseases" group)
**C**omparator (control group of non-exposed people)	Patients without periodontitis (i.e., those with clinically healthy periodontium, gingivitis, or a combination of patients with these conditions)	— Lack of non-periodontitis group<br>— Inadequate control group: control group that includes patients with stage I/mild or localized periodontitis
**O**utcome	— Oral Health-Related Quality of Life (OHRQoL), as assessed by the Oral Health Impact Profile (OHIP-14), in both the periodontitis group and the control group, or the difference between these groups<br>— At least one of the following must be reported (or data that allows for its calculation must be available):<br>- severity of impacts: the mean OHIP-14 total score (the sum of ordinal responses) along with the standard deviation (SD)<br>- prevalence of impacts: the percentage of study participants reporting 'fairly often' or 'very often' on one or more items<br>- extent of impacts: the mean (SD) total number of items reported "fairly often" or "very often" by study participants	— OHRQoL tool other than OHIP-14, including other OHIP versions (e.g. S-OHIP, OHIP-CP) and other questionnaires (e.g. GOHAI)<br>— Adequate OHIP data not reported and cannot be calculated (e.g. publications reporting only median values, p values of between-group differences or regression analysis coefficients)<br>— OHIP data reported without division into adequate periodontitis and non-periodontitis groups

PECOS	Inclusion criteria	Exclusion criteria
**S**tudy design	Cohort, case-control or cross-sectional study	— Non-primary research publication (review, comment, editorial, opinion)<br>— Interventional study (in cases of interventional studies including non-periodontitis cohort/arm, the inclusion of baseline OHIP-14 data, treated as cross-sectional data, were considered*)<br>— Case series<br>— Case report<br>— Expert panel
Other	Studies published in English or Polish	Meeting abstracts and posters<br>Results obtained from overlapping populations (in cases where studies conducted on overlapping populations were identified, the results from the larger population were included in the meta-analysis)^

* eventually no interventional study was eligible for inclusion; ^ no publication was excluded for this reason

Table S5. Quality of the included studies (risk-of-bias), based on the Quality Assessment Tool for Observational Cohort and Cross-Sectional Studies developed by NHLBI (1)

**Criteria (exposure: periodontitis; outcome: OHRQoL measured with OHIP-14)** **Response options: Yes/No/CD/NA/NR**	***Al Habashneh 2012 (2)***	***Botelho 2020 (3)***	***Cataldo 2024 (4)***	***Dikilitaş 2024 (5)***	***Eroğlu 2023 (6)***	***Fuller 2020^ (7)***	***Mishra 2023† (8)***	***Santonocito 2021 (9)***	***Ustaoğlu 2019*** $^{£}$ ***(10)***
1. Was the research question or objective in this paper clearly stated?	Yes	Yes	Yes	Yes	Yes	Yes	Yes	Yes	Yes
2. Was the study population clearly specified and defined?	Yes	Yes	Yes	Yes	Yes	Yes	Yes	No‡	Yes
3. Was the participation rate of eligible persons at least 50%?	CD	CD	CD	CD	CD	CD	CD	CD	CD
4. Were all the subjects selected or recruited from the same or similar populations (including the same time period)? Were inclusion and exclusion criteria for being in the study prespecified and applied uniformly to all participants?	Yes	Yes	Yes	Yes	Yes	Yes	Yes	CD	Yes
5. Was a sample size justification, power description, or variance and effect estimates provided?	No	No*	Yes	Yes	Yes	No	Yes	Yes	Yes
6. For the analyses in this paper, were the exposure(s) of interest measured prior to the outcome(s) being measured?	CD	CD	CD	Yes	CD	No	No	Yes	Yes
7. Was the timeframe sufficient so that one could reasonably expect to see an association between exposure and outcome if it existed?	CD	CD	CD	CD	CD	CD	CD	CD	CD
8. For exposures that can vary in amount or level, did the study examine different levels of the exposure as related to the outcome (e.g., categories of exposure, or exposure measured as continuous variable)?	Yes	Yes	No	No	Yes	Yes	No	No	No
9. Were the exposure measures (independent variables) clearly defined, valid, reliable, and implemented consistently across all study participants?	Yes	Yes	Yes	Yes	Yes	Yes	Yes	CD	Yes
10. Was the exposure(s) assessed more than once over time?	NA	NA	NA	NA	NA	NA	NA	NA	NA
11. Were the outcome measures (dependent variables) clearly defined, valid, reliable, and implemented consistently across all study participants?	Yes	Yes	Yes	Yes	Yes	Yes	Yes	Yes	Yes
12. Were the outcome assessors blinded to the exposure status of participants?	CD	CD	CD	Yes	CD	Yes	CD	CD	Yes
13. Was loss to follow-up after baseline 20% or less?	NA	NA	NA	NA	NA	NA	NA	NA	NA
14. Were key potential confounding variables measured and adjusted statistically for their impact on the relationship between exposure(s) and outcome(s)?	Yes	No	Yes	No	No	No	No	Yes	No
**QUALITY RATING (based on the nb of "Yes" answers: 0-4 - Poor, 5-9 - Fair, 10-14 - Good)**	**Fair**	**Fair**	**Fair**	**Fair**	**Fair**	**Fair**	**Fair**	**Fair**	**Fair**

Abbreviations: CD – cannot determine; NA – not applicable; NR – not reported

* sample size justification, given in the separate paper (11), pertained to the broader sample than *Botelho 2020* analysis (3); ^ only groups defined according to AAP 2007 and analyses pertaining to those groups were considered; † only "PD" and "Healthy" groups were considered; ‡ source population of the control group is unclear and reported mean age is inconsistent with the eligibility criteria; $^{£}$ only "Chronic periodontitis" and "Gingivitis" groups were considered

Table S6. Detailed characteristics and outcomes of the included studies

Study (country)	Design, sampling	Study population	Details of the clinical examination and OHRQoL assessment	Periodontitis group/s	Non-periodontits group/s	Reported outcomes of OHIP-14 assessment in periodontitis and control groups:<br>1) Severity of impacts<br>2) Prevalence of impacts<br>3) Extent of impacts
Al Habashneh 2012 (2)<br>(Jordan)	Cross-sectional;<br>systematic random sample	Source population: patients referred to dental clinics at the Department of Periodontology at the Dental Teaching Center of Jordan University of Science and Technology in Irbid, Jordan; university students, employees, and their families who are insured, the general population of north Jordan, employees of the government.<br>Inclusion criteria:<br>• 18 years or older<br>• at least 15 teeth present<br>Exclusion criteria:<br>• presence of a mental or psychological disorder or use of antipsychotic medication<br>• need for antibiotic coverage during routine dental procedures<br>• presence of removable dentures<br>• presence of carious lesions or symptomatic oral lesions	Examiner calibration: unclear<br>Examined teeth: all teeth, excluding third molars<br>Examined sites per tooth: 4 (mesiofacial, midfacial, distofacial, mesiolingual, midlingual, distolingual)<br>OHIP-14 administration: personal interview<br>OHIP-14 version: Arabic, linguistically and culturally adapted for the purpose of the study, no previous validation<br>OHIP-14 data validation: Y (face validity, content validity, comprehensiveness, test-retest reliability, item internal consistency, item	Periodontitis was defined as the presence of four or more teeth with one site or more with PPD ≥4 mm and CAL ≥3 mm.<br>The severity of chronic periodontitis was classified as mild (attachment loss of 1 to 2 mm), moderate (attachment loss of 3 to 4 mm), and severe (attachment loss of 5 mm or more) [according to (12)].<br>N = 233 (79 mild / 93 moderate / 61 severe)	Chronic gingivitis<br>N = 167	1) Mean (SD) total OHIP-14 score in gingivitis, mild periodontitis, moderate periodontitis and severe periodontitis groups: 9.53 (7.12), 8.93 (6.61), 12.55 (7.35), 15.57 (7.49). The average OHIP-14 score was significantly ($p < 0,05$) higher in patients with severe or moderate periodontitis than in patients with chronic gingivitis or mild periodontitis (the comparison was adjusted for age, sex, years of education, medical illnesses, smoking status, and family income; the test not reported).<br>2) FOVO was reported on ≥1 items by 32,9% of patients with gingivitis, 31.6% patients with mild periodontitis, 53.8% patients with moderate periodontitis and 63.9% patients with severe periodontitis.<br>3) NR

Study (country)	Design, sampling	Study population	Details of the clinical examination and OHRQoL assessment	Periodontitis group/s	Non-periodontits group/s	Reported outcomes of OHIP-14 assessment in periodontitis and control groups:<br>1) Severity of impacts<br>2) Prevalence of impacts<br>3) Extent of impacts
			discriminant validity, internal consistency [Cronbach's alpha ≥0.70])			
Botelho 2020 (3) [SoPHiAS substudy]<br>(Portugal)	Cross-sectional; random sample, geographically stratified	Source population: the Study of Periodontal Health in Almada-Seixal (SoPHiAS), a population-based representative study, with a target population living in the municipalities of Almada and Seixal (Portugal). In the *Botelho 2020* a subset of participants of the SoPHiAS study with 65 years old or over was studied.<br>Inclusion criteria: 65 years or over<br>Exclusion criteria: none	Examiner calibration: Y<br>Examined teeth: all fully erupted teeth, excluding third molars, implants and retained roots<br>Examined sites per tooth: 6 (mesiobuccal, buccal, distobuccal, mesiolingual, lingual, and distolingual)<br>OHIP-14 administration: self-administered<br>OHIP-14 version: Portuguese, previously validated (13)<br>OHIP-14 data validation: N	Cases of periodontitis according to the AAP/EFP consensus (2017): interdental CAL ≥2 non-adjacent teeth, or buccal or oral CAL ≥3 mm with PPD >3 mm is detectable at ≥2 teeth.<br>Staging according to severity and extent (AAP/EFP consensus, 2017). For the severity, interdental CAL at site of greatest loss of 1–2 mm, 3–4 and ≥5 was considered as mild (stage 1), moderate (stage 2), and severe (stage 3 and stage 4), respectively. For the extent, a case was described as localized (<30% of teeth involved), generalized (≥30% of	Patients categorised as "healthy" in the assessment of the severity and as "no" in the assessment of the extent of periodontitis.<br>N = 172	1) Mean (SD) total OHIP-14 score in "no disease", "localized periodontitis" and "generalized periodontitis" groups: 7.15 (10.35), 7.51 (9.81), 8.42 (10.98); p = 0,178 (Kruskal-Wallis test). Mean (SD) total OHIP-14 score in stage "0 (healthy)", "1 (mild periodontitis)", "2 (moderate periodontitis)" and "3 (severe/advanced periodontitis)" groups: 7.2 (10.3), 6.3 (8.9), 7.1 (9.2), 9.9 (12.2); p = 0,225 (Kruskal-Wallis test).<br>2) NR<br>3) NR

Study (country)	Design, sampling	Study population	Details of the clinical examination and OHRQoL assessment	Periodontitis group/s	Non-periodontits group/s	Reported outcomes of OHIP-14 assessment in periodontitis and control groups:<br>1) Severity of impacts<br>2) Prevalence of impacts<br>3) Extent of impacts
				teeth involved) or molar/incisor pattern.<br>N = 420 (142 localized / 278 generalized; 84 stage I, 156 stage II, 180 stage III)		
Cataldo 2024 (4) (Brazil)	Case-control; random sample	<u>Source population</u>: individuals who attended a Dental School Clinic; periodontitis patients were randomly selected at the Periodontics and Dentistry Clinics.<br><u>Inclusion criteria</u>: 35-70 years<br><u>Exclusion criteria</u>:<br>• smoking<br>• presence of diabetes, mental disorders or any systemic conditions<br>• periodontal treatment for at least 1 year<br>• pregnancy, lactation<br>• use of prostheses and/or orthodontic appliances<br>• treatment with anti-depressive or undergoing psychological treatment, antibiotic, or anti-inflammatory medication for at least 6 months	<u>Examiner calibration</u>: Y<br><u>Examined teeth</u>: full-mouth examination, further details not reported<br><u>Examined sites per tooth</u>: 6<br><u>OHIP-14 administration</u>: unclear<br><u>OHIP-14 version</u>: Brazilian, previously validated (14)<br><u>OHIP-14 data validation</u>: N	Presence of 2 or more teeth with at least 1 observable buccal or interproximal site with a clinical attachment loss (CAL) ≥ 3 mm and a periodontal probing depth (PPD) > 3 mm. Bone loss was confirmed by radiographic examination.<br>N = 50	<10% of bleeding sites with probing depths ≤3 mm and absence of detectable attachment and/or bone loss ("gingival health" according to Chapple 2018).<br>N = 50	1) Mean (SD) total OHIP-14 score in "periodontitis" and "control" group: 19.1 (11.2), 7.8 (7.3). The OHIP-14 total score demonstrated a significant positive association with periodontitis in the unadjusted univariate logistic regression analysis (OR = 1.15; $p < 0.001$; 95% CI: 1.08–1.22) and in the adjusted multiple binary logistic regression (OR = 1.17; $p < 0.0001$; 95% CI: 1.08–1.226; controlled for age, education and anxiety).<br>2) NR<br>3) NR

Study (country)	Design, sampling	Study population	Details of the clinical examination and OHRQoL assessment	Periodontitis group/s	Non-periodontits group/s	Reported outcomes of OHIP-14 assessment in periodontitis and control groups:<br>1) Severity of impacts<br>2) Prevalence of impacts<br>3) Extent of impacts
Dikilitaş 2024 (5) (Turkey)	Cross-sectional; sampling method not described	Source population: individuals selected from the patients who applied to Usak University Oral and Maxillofacial Radiology clinic for routine clinical examination.<br>Inclusion criteria:<br>• 18 years or older<br>• systemically good health<br>Exclusion criteria:<br>• current and former smokers<br>• diabetes<br>• cardiovascular or immunological diseases<br>• individuals who took any drug that could affect periodontal status	Examiner calibration: Y<br>Examined teeth: all teeth<br>Examined sites per tooth: 6<br>OHIP-14 administration: self-administered<br>OHIP-14 version: Turkish, previously validated (15)<br>OHIP-14 data validation: N	Stage I periodontitis: Interdental CAL 1–2 mm, radiographic bone loss <15%, PPD ≤4 mm and no tooth loss due to periodontitis (according to (16)).<br>N = 36	Three non-periodontits groups (according to (17)):<br>1) clinical periodontal health with intact periodontium: BOP <10%, PPD ≤3 mm, no attachment or radiographic bone loss (N = 44)<br>2) clinical periodontal health with reduced periodontium: PPD ≤3 mm, or PPD ≤4 mm with no site ≥4 mm with bleeding on probing (N = 42)<br>3) gingivitis: BOP score ≥10% and no attachment or radiographic bone loss (N = 44)<br>Total N = 130	1) Mean (SD) total OHIP-14 score in “clinical periodontal health with intact periodontium”, “clinical periodontal health with reduced periodontium”, “gingivitis” and “stage I periodontitis” groups: 1.09 (1.58), 2.95 (2.58), 11.61 (3.21), 13.03 (3.47). Statistically significant differences (p < 0,001; the test not reported): “clinical periodontal health with intact periodontium” versus “clinical periodontal health with reduced periodontium”, “gingivitis” and “stage I periodontitis”; “clinical periodontal health with reduced periodontium” versus “gingivitis” and “stage I periodontitis”.<br>2) NR<br>3) NR

Study (country)	Design, sampling	Study population	Details of the clinical examination and OHRQoL assessment	Periodontitis group/s	Non-periodontonts group/s	Reported outcomes of OHIP-14 assessment in periodontitis and control groups:<br>1) Severity of impacts<br>2) Prevalence of impacts<br>3) Extent of impacts
Eroğlu 2023 (6) (Turkey)	Cross-sectional; random sample	Source population: individuals selected from among individuals who presented to Necmettin Erbakan University Faculty of Dentistry Department of Periodontology.<br>Inclusion criteria:<br>• 18-65 years<br>• diagnosis of healthy gingiva or periodontitis (according to (18))<br>Exclusion criteria:<br>• sleep apnea<br>• <20 teeth (excluding third molars and retained roots)<br>• periodontal treatment in the last year<br>• acute dental conditions that required urgent care, including abscesses and cellulitis, or diseases impacting the jawbones, such as cysts and neoplasms<br>• concomitant medical conditions (e.g., diabetes, cardiovascular diseases, hypertension, or hypercholesterolemia) or active infectious/ inflammatory diseases (e.g., HIV, hepatitis, tuberculosis, rheumatoid arthritis, allergies, or asthma)<br>• pregnant or lactating females	Examiner calibration: Y<br>Examined teeth: all teeth except for the third molars<br>Examined sites per tooth: 6<br>OHIP-14 administration: self-administered<br>OHIP-14 version: Turkish, previously validated (15)<br>OHIP-14 data validation: Y (internal consistency [Cronbach's alpha ≥0.70])	Periodontitis: the detection of interdental clinical attachment loss (CAL) in ≥2 non-adjacent teeth or buccal CAL of more than 3 mm with a probing depth of ≥3 mm in ≥2 teeth (according to 2017 World Workshop on the Classification of Periodontal and Peri-Implant Diseases and Conditions, (16)).<br>The diagnosed periodontitis was classified on the basis of the stage and grade system (16).<br>N = 93 (13 stage I / 31 stage II / 37 stage III / 12 stage IV; 7 grade A / 42 grade B / 44 grade C)	Clinical gingival health: ≤3 mm probing depth and <10% bleeding site (according to 2017 World Workshop on the Classification of Periodontal and Peri-Implant Diseases and Conditions, (17)).<br>N = 31	1) Mean (SD) total OHIP-14 score in "gingival healthy" and "periodontitis" groups: 7,71 (9,01) and 11,39 (13,15); p = 0,038 (independent sample t-test). Mean (SD) total OHIP-14 score by periodontitis stage: "gingival healthy" - 7,71 (9,01) / stage I - 13.00 (16.51) / stage II - 10.39 (12.30) / stage III - 10.78 (12.55) / stage IV - 14.17 (12.52); p = 0,514 (one-way ANOVA test). Mean (SD) total OHIP-14 score by periodontitis grade: "gingival healthy" - 7,71 (9,01) / grade A - 15.14 (18.99) / grade B - 10.05 (13.33) / grade C - 12.27 (11.63); p = 0,306 (one-way ANOVA test).<br>2) NR<br>3) NR

Study (country)	Design, sampling	Study population	Details of the clinical examination and OHRQoL assessment	Periodontitis group/s	Non-periodontits group/s	Reported outcomes of OHIP-14 assessment in periodontitis and control groups:<br>1) Severity of impacts<br>2) Prevalence of impacts<br>3) Extent of impacts
		• malignancy<br>• treatment with systemic antibiotics, corticosteroids and/or immunosuppressive agents within 3 months prior to periodontal examination<br>• psychiatric, mental or physical disability<br>• depression and accordingly used antidepressants				
Fuller 2020 (7) (United Kingdom)	Case-control; sampling method not described	Source population: Cases were recruited from patients referred to the Eastman Dental Hospital, University College London by general dental practitioners. Periodontally healthy controls were recruited among patients referred to other Departments of the same hospital.<br>Inclusion criteria: not stated<br>Exclusion criteria:<br>• known systemic diseases (cardiovascular, respiratory, renal, malignancy, etc)<br>• history and/or presence of any other infections<br>• systemic antibiotic treatment in the preceding 3 months<br>• long-term treatment with any medication suspected to affect the periodontium (eg non-	Examiner calibration: Y<br>Examined teeth: full-mouth examination, further details not reported<br>Examined sites per tooth: 6<br>OHIP-14 administration: unclear<br>OHIP-14 version: original (English), previously validated (19)<br>OHIP-14 data validation: unclear	1) Mild periodontitis, according to AAP 2007 definition (20, 21), N = 93<br>2) Moderate-to-severe periodontitis, according to AAP 2007 definition (21), N = 240<br>Total N = 333	"No periodontitis" according to AAP 2007 (21)<br>N = 138	1) Mean (SD) total OHIP-14 score in "no perio", "mild perio" and "moderate to severe perio" groups: 5.20 (6.62), 8.94 (7.61), 14.89 (10.76); p < 0,001 (ANOVA).<br>2) FOVO reported on ≥1 item in "no perio", "mild perio" and "moderate to severe perio" groups [n (%)]:12 (8.7%), 20 (21.5%), 129 (53.8%); p < 0,001 (chi-square test).<br>3) NR

**Study (country)**	**Design, sampling**	**Study population**	**Details of the clinical examination and OHRQoL assessment**	**Periodontitis group/s**	**Non-periodontits group/s**	**Reported outcomes of OHIP-14 assessment in periodontitis and control groups:** 1) **Severity of impacts** 2) **Prevalence of impacts** 3) **Extent of impacts**
		steroidal anti-inflammatory drugs)<br>• pregnant or lactating females<br>• <20 teeth present				
Mishra 2023 (8) (India)	Cross-sectional; sampling method not described	Source population: individuals recruited from Maitri College of Dentistry and Research Center (MCDRC), Anjora, Durg, Chhattisgarh, India.<br>Inclusion criteria:<br>• at least 20 teeth excluding the third molars<br>• patients with generalized periodontitis or periodontally healthy<br>Exclusion criteria:<br>• any coexisting systemic diseases or inflammatory conditions<br>• periodontal therapy or antibiotic/anti-inflammatory therapy for the past six months prior to the examination<br>• pregnant women, and lactating mothers	Examiner calibration: Y<br>Examined teeth: all teeth except for the third molars<br>Examined sites per tooth: 6 (mesiobuccal, mid-buccal, distobuccal, distolingual/palatal, mid-lingual/palatal, and mesiolingual/palatal)<br>OHIP-14 administration: self-administered<br>OHIP-14 version: Hindi, previously validated (22)<br>OHIP-14 data validation: Y (internal consistency [Cronbach's alpha ≥0.70]; the expected	Generalized stage II/III/IV periodontitis (according to the World Workshop on the Classification of Periodontal and Peri-Implant Diseases and Conditions 2017 criteria, (18))<br>N = 50	Periodontally healthy controls<br>N = 50	1) Mean (SD) total OHIP-14 score in "periodontits" and "healthy" group: 17.02 (9.99) and 6.32 (5.59); significantly different by the Post-Hoc Dunn's test using a Bonferroni corrected alpha of 0.0083.<br>2) NR<br>3) NR

Study (country)	Design, sampling	Study population	Details of the clinical examination and OHRQoL assessment	Periodontitis group/s	Non-periodontits group/s	Reported outcomes of OHIP-14 assessment in periodontitis and control groups:<br>1) Severity of impacts<br>2) Prevalence of impacts<br>3) Extent of impacts
			set of four dimensions [oral function, orofacial appearance, orofacial pain and psychosocial impact] was identified)			
Santonocito 2021 (9) (Italy)	Case-control, matched controls (age, gender); sampling method not described	Source population: patients with periodontitis who referred to the School of Dentistry at the University of Catania, Italy and matched controls.<br>Inclusion criteria:<br>• 18-50 years<br>• ≥20 teeth present<br>Exclusion criteria:<br>• psychiatric disorders<br>• drug abuse<br>• taking sedative, anxiolytic or analgesic drugs up to a week earlier<br>• pregnancy or breastfeeding<br>• acute dental or periodontal condition	Examiner calibration: Y<br>Examined teeth: each tooth, further details not reported<br>Examined sites per tooth: 6<br>OHIP-14 administration: face-to-face interview<br>OHIP-14 version: Italian, previously validated (23)<br>OHIP-14 data validation: N	Periodontitis, according to EFP/AAP 2017 consensus definition*<br>N = 55*	Patients matched for age and gender to periodontitis group, with no history of periodontal disease<br>N = 56*	1) Mean (SD) total OHIP-14 score in "periodontits" and "control" groups: 11.89 (2.5) and 6.89 (2.1); p = 0,001 (t-test). Periodontitis was a significant risk factor for higher OHIP global scores in the multivariate linear regression analysis (p = 0.006, β = -4.044, SE = 1.478, β = -0.254, 95% confidence interval -6.442 to -1.235; controlled for schooling years, smoking habits, pain in Numerical Rating Scale, periodontal indexes, number of missing teeth).<br>2) NR<br>3) NR
Ustaoğlu 2019 (10) (Turkey)	Cross-sectional; sampling method not described	Source population: individuals recruited from the patient pool of the Department of Periodontology at the University of Bolu Abant İzzet Baysal.	Examiner calibration: Y	1) Generalized chronic periodontitis: cases who has 4 or more than 4 teeth in each jaw with PD of	Gingivitis: cases with BOP at equal or more than 20% of the sites and GI ≥1, with no sites with PD and	1) Mean (SD) total OHIP-14 score in "generalized chronic periodontits" and "gingivitis" groups: 13,53 (9.38) and 7.06 (5.03).

Study (country)	Design, sampling	Study population	Details of the clinical examination and OHRQoL assessment	Periodontitis group/s	Non-periodontits group/s	Reported outcomes of OHIP-14 assessment in periodontitis and control groups: 1) Severity of impacts 2) Prevalence of impacts 3) Extent of impacts
		Inclusion criteria:<br>• ability to understand verbal or written instructions<br>• no use of systemic medications (ie, muscle relaxants, anti-inflammatory medications, sedatives and narcotic analgesics) within the past 3 months<br>Exclusion criteria:<br>• pregnancy or lactation<br>• age <18 years<br>• systemic diseases that influence periodontal tissues	Examined teeth: all teeth except third molars<br>Examined sites per tooth: 6<br>OHIP-14 administration: face-to-face interview<br>OHIP-14 version: Turkish, no reference given, information on previous validation lacking<br>OHIP-14 data validation: Y (internal consistency [Cronbach's alpha ≥0.70])	≥5 mm, CAL of ≥4 mm, BOP at more than 80% of the proximal sites and radiographic evidence of interproximal bone loss<br>N = 114<br>2) Generalized aggressive periodontitis: patients less than 35 years of age, with more than 20 teeth, have more than 8 teeth with a PD of more than 5 mm (three of teeth must be other than first molar or incisor), with CAL of more than 3 mm, and the cases where clinical diagnosis was confirmed by the presence of interproximal bone loss on radiographic examination<br>N = 100^	CAL more than 3 mm or bone loss<br>N = 109	2) NR<br>3) NR

Abbreviations: AAP - American Academy of Periodontology; BOP - bleeding on probing; CAL – clinical attachment loss; CI – confidence interval; EFP - European Federation of Periodontology; FOVO – "fairly often" or "very often"; N – no; NR – not reported; OR – odds ratio; PPD – probing pocket depth; SD – standard deviation; SE – standard error; Y - yes

^ generalized aggressive periodontits group was not included in the meta-analyses; * due to inconsistencies in the publication, the information was confirmed through personal communication with the corresponding author

Table S7. Results of the univariate and bivariate meta-regression analyses for the mean difference in total OHIP score (periodontitis versus controls), random model

Analysis	Parameter	No of studies	Point estimate (β coefficient)	Standard error	Z-value	p-value (2-sided)	95% confidence interval
Univariate: age	Intercept	8	11.468	3.5691	3.213		4.473; 18.463
	Mean age (sample) [years]		-0.115	0.0764	-1.503	0.133	-0.265; 0.035
Univariate: age difference	Intercept	5	7.35	1.8139	4.052		3.795; 10.905
	Mean difference in age (P vs C) [years]		0.079	0.2076	0.382	0.703	-0.328; 0.486
Bivariate: age, age difference	Intercept	5	3.952	10.9669	0.36		-17.543; 25.446
	Mean age (sample) [years]		0.076	0.2283	0.334	0.738	-0.371; 0.524
	Mean difference in age (P vs C) [years]		0.126	0.3205	0.393	0.694	-0.502; 0.754
Univariate: sex	Intercept	8	-0.431	8.4141	-0.051		-16.922; 16.061
	Mean percentage of females (sample)		0.114	0.1493	0.766	0.444	-0.178; 0.407
Univariate: sex difference	Intercept	6	6.42	0.832	7.717		4.79; 8.051
	Mean difference in percentage of females (P vs C)		0.337	0.0944	3.575	**<0.001**	0.152; 0.522
Bivariate: sex, sex difference	Intercept	6	7.019	6.3461	1.106		-5.419; 19.457
	Mean percentage of females (sample)		-0.009	0.115	-0.082	0.935	-0.235; 0.216
	Mean difference in percentage of females (P vs C)		0.335	0.1177	2.844	**0.004**	0.104; 0.565
Univariate: PPD	Intercept	9	-2.7	2.7644	-0.977		-8.118; 2.718
	Mean PPD (sample) [mm]		3.088	0.9467	3.262	**0.001**	1.233; 4.944
Univariate: PPD difference	Intercept	5	0.23	1.8581	0.124		-3.412; 3.872
	Mean difference in PPD (P vs C) [mm]		3.741	1.0105	3.702	**<0.001**	1.76; 5.721
Bivariate: PPD, PPD difference	Intercept	5	3.044	4.5994	0.662		-5.971; 12.059
	Mean PPD (sample) [mm]		-1.274	1.9021	-0.67	0.503	-5.003; 2.454
	Mean difference in PPD (P vs C) [mm]		4.426	1.4586	3.034	**0.002**	1.567; 7.284

Abbreviations: C – control group; no – number; P – periodontitis group; PPD – probing pocket depth

Figure S1 . Risk-of-bias traffic plot, based on the Quality Assessment Tool for Observational Cohort and Cross-Sectional Studies developed by NHLBI (1)

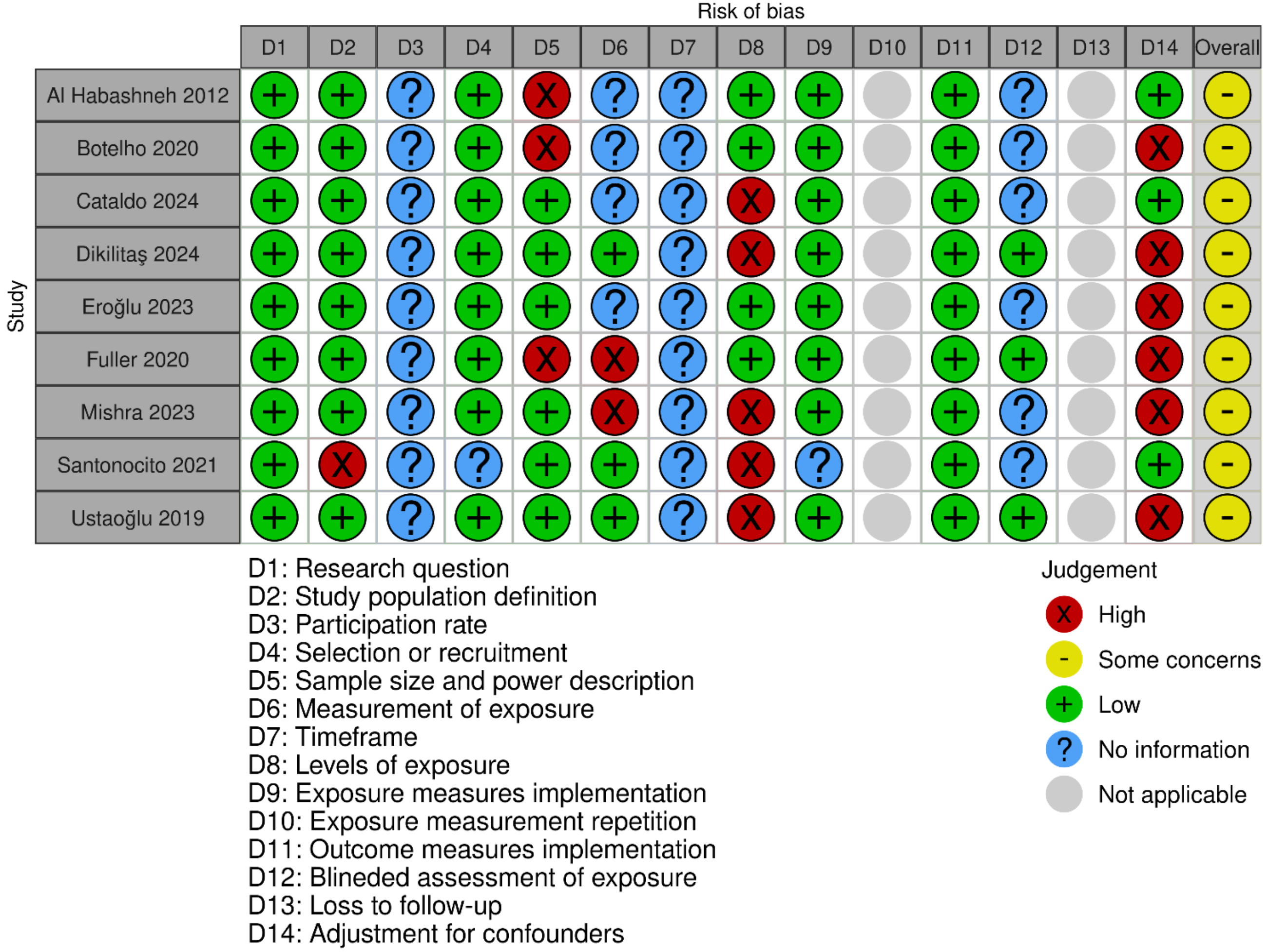

The risk-of-bias plot was generated using the *robvis* tool (24)

Figure S2 . Risk of bias due to missing results (funnel plot)

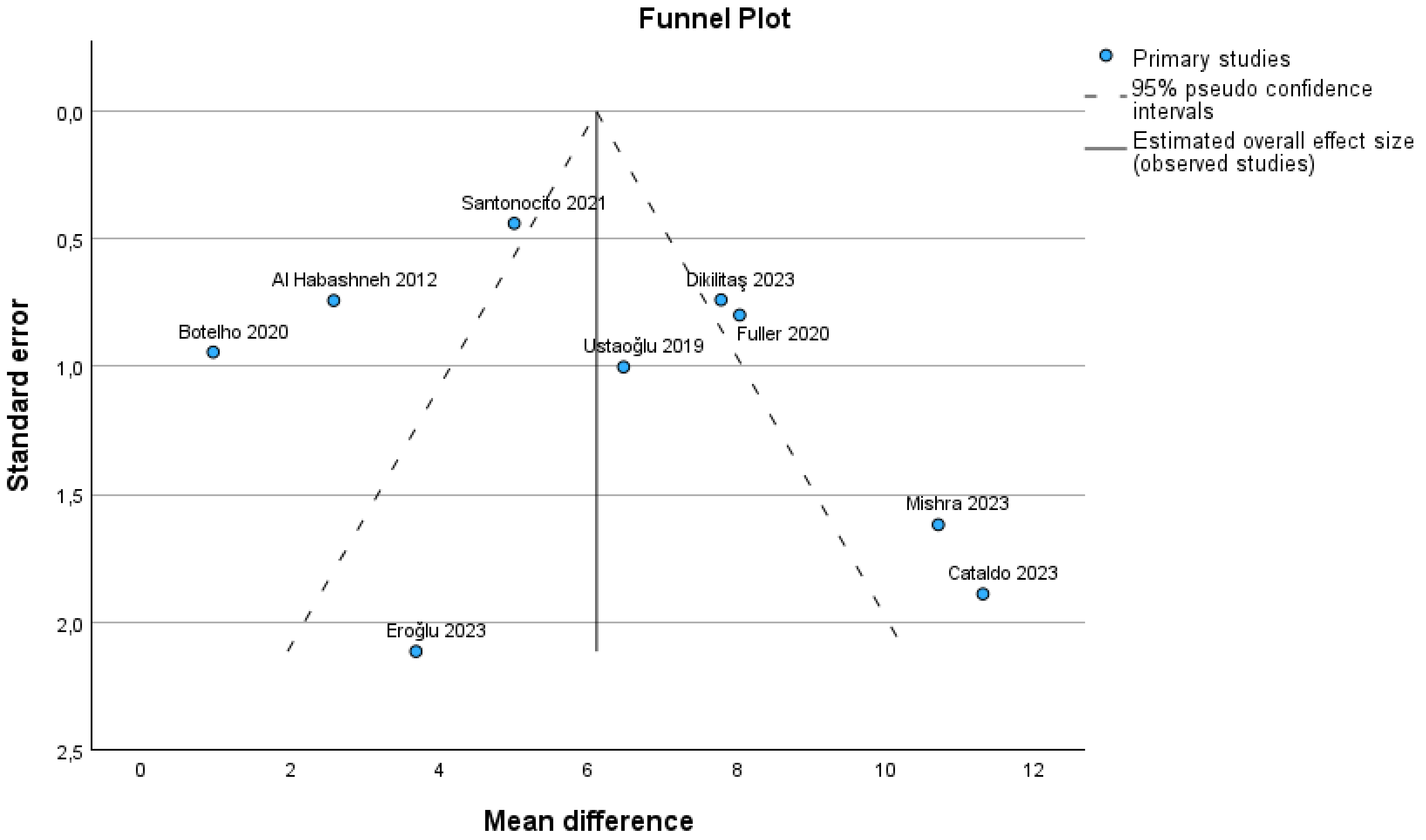

Egger's test for publication bias: bias coefficient = 2.276889 (95% CI = -2.945993 - 7.499771), P = 0.3369

Figure S3. Weighted mean difference in severity of impacts (mean OHIP-14 total score) in periodontitis patients compared to control subjects, meta-analyses in subgroups of studies by the difference in mean age between P and C groups; only studies with available mean age for P and C groups were included

Study	Age difference (P vs C) [y]	N Periodontitis	N Controls	Weight [%]	MD (95% CI)
<u>**Mean age in P group higher by >10 years**</u>					
Ustaoğlu 2019	15,5	114	109	78.1	6.47 (4.51; 8.43)
Cataldo 2024	11,6	50	50	21.9	11.30 (7.59; 15.01)
**WMD (95% CI) [fixed; $p = 0.0240$, $I^2 = 80.4\%$]**		**164**	**159**	**n/a**	**7.53 (5.79; 9.26),** p < 0.0001
<u>**Mean age in P group higher by 1-5 years**</u>					
Dikilitaş 2024 (P vs pooled C)	3,59	36	130	82.8	7.78 (6.33; 9.23)
Mishra 2023	3,37	50	50	17.2	10.70 (7.53; 13.87)
**WMD (95% CI) [fixed; $p = 0.1006$, $I^2 = 62.9\%$]**		**86**	**180**	**n/a**	**8.28 (6.96; 9.60),** p < 0.0001
<u>**Mean age in P group higher by <1 year**</u>					
Santonocito 2021	0,6	55	56	100	5.00 (4.14; 5.86), p < 0,0001
<u>**Total**</u>					
**WMD (95% CI) [random; $p < 0.0001$, $I^2 = 85.3\%$]**		**305**	**395**	**n/a**	**7.80 (5.67; 9.93),** p < 0.0001

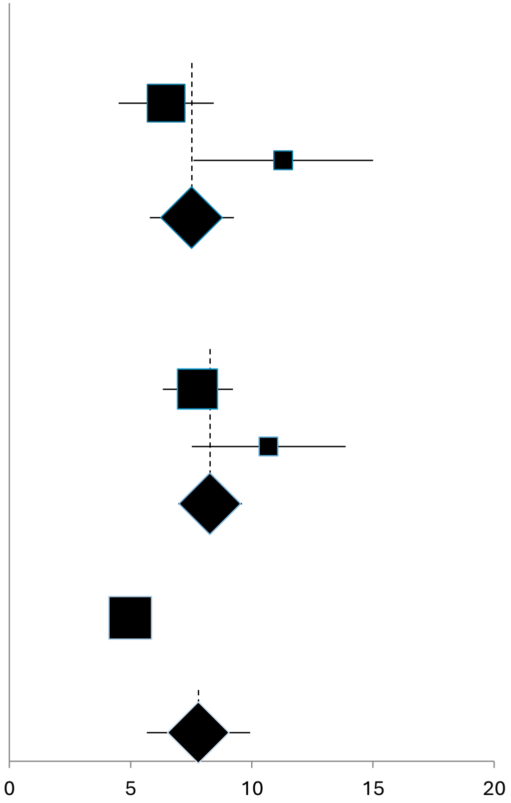

WMD – weighted mean difference; MD – mean difference; CI – confidence interval; N/A – not applicable; P – periodontitis; C – control

Figure S4 . Prediction line determined by meta-regression for difference in mean age (periodontitis group versus control group) as an independent variable

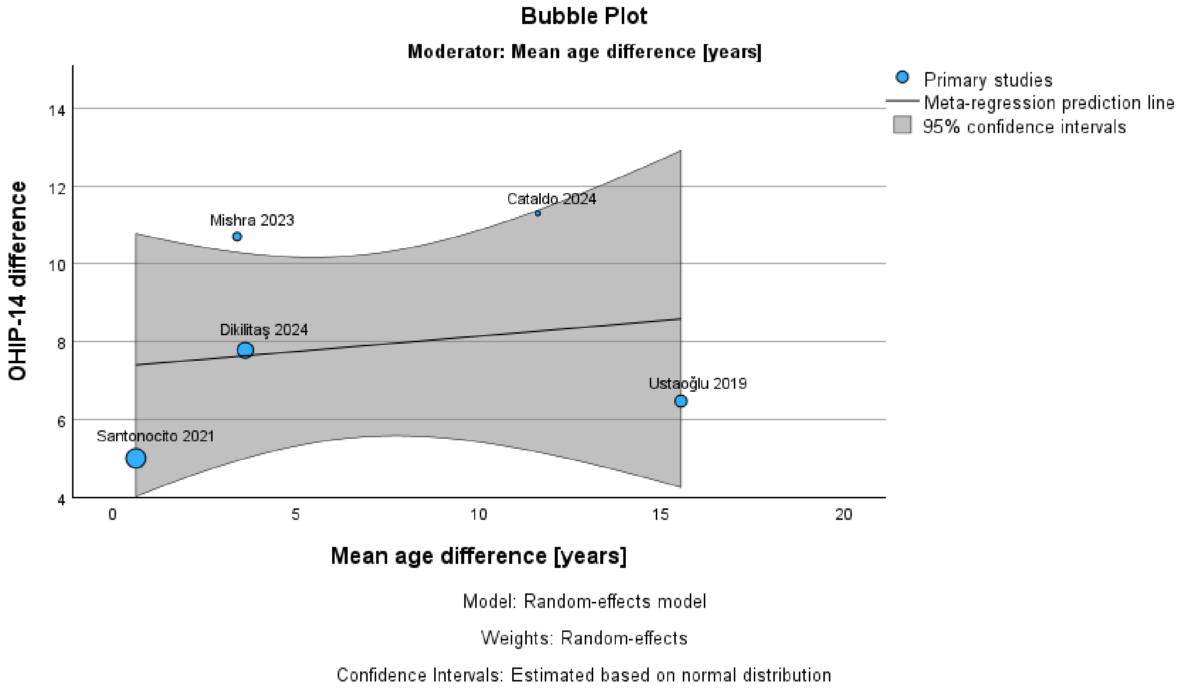

Figure S5 . Prediction line determined by meta-regression for mean age of the sample as an independent variable

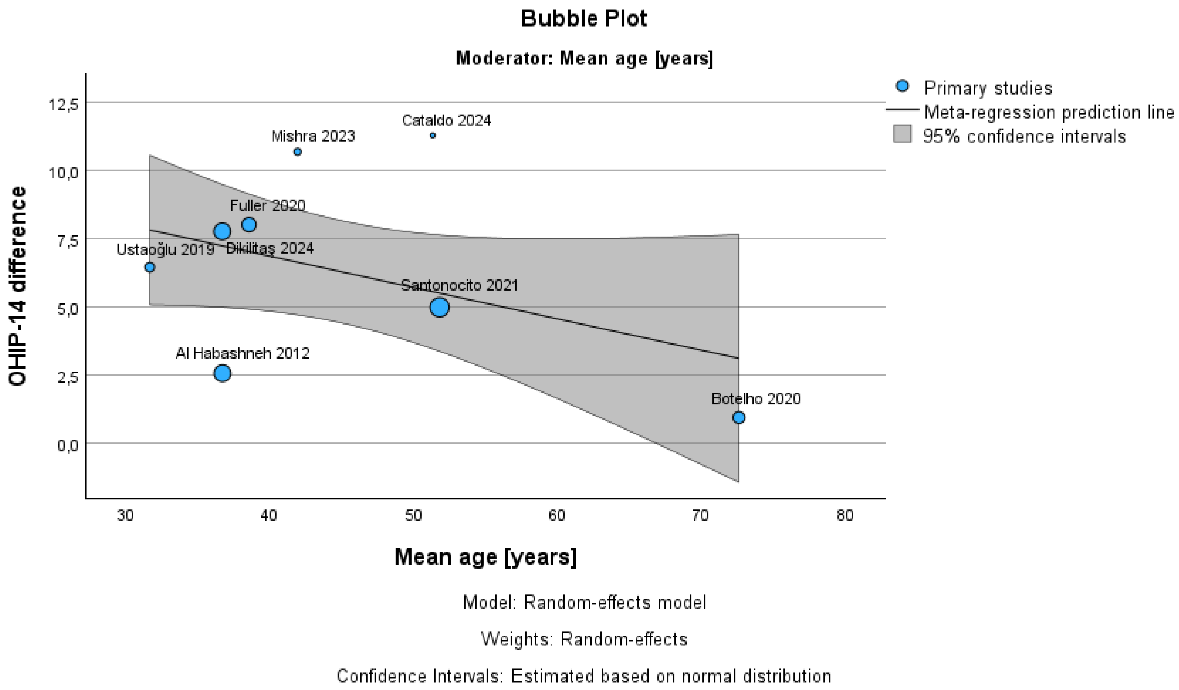

Figure S6 . Weighted mean difference in severity of impacts (mean OHIP-14 total score) in periodontitis patients compared to control subjects, meta-analyses in subgroups of studies by group with the higher percentage of females; only studies with available percentage of females for P and C groups were included

Study	Diff in % of fem (P vs C)	N Periodontitis	N Controls	Weight [%]	MD (95% CI)
<u>Percentage of females higher in P vs C group</u>					
Cataldo 2024	14,0%	50	50	16.9	11.30 (7.59; 15.01)
Mishra 2023	4,0%	50	50	23	10.70 (7.53; 13.87)
Ustaoğlu 2019	3,9%	114	109	60.1	6.47 (4.51; 8.43)
**WMD (95% CI) [fixed; $p = 0.0179$, $I^2 = 75.2\%$]**		**214**	**209**	**n/a**	**8.26 (6.74; 9.78),** $p < 0.0001$
<u>Percentage of females higher in C versus P group</u>					
Santonocito 2021	-4,6%	55	56	40.2	5.00 (4.14; 5.86)
Botelho 2020	-9,0%	420	172	36.1	0.96 (-0.89; 2.81)
Eroğlu 2023	-17,2%	93	31	23.7	3.68 (-0.47; 7.83)
**WMD (95% CI) [random; $p = 0.0005$, $I^2 = 86.8\%$]**		**513**	**203**	**n/a**	**3.23 (0.15; 6.31),** $p = 0.0396$
<u>Total</u>					
**WMD (95% CI) [random; $p = 0.0005$, $I^2 = 86.8\%$]**		**727**	**412**	**n/a**	**6.14 (3.55; 8.73),** $p < 0.0001$

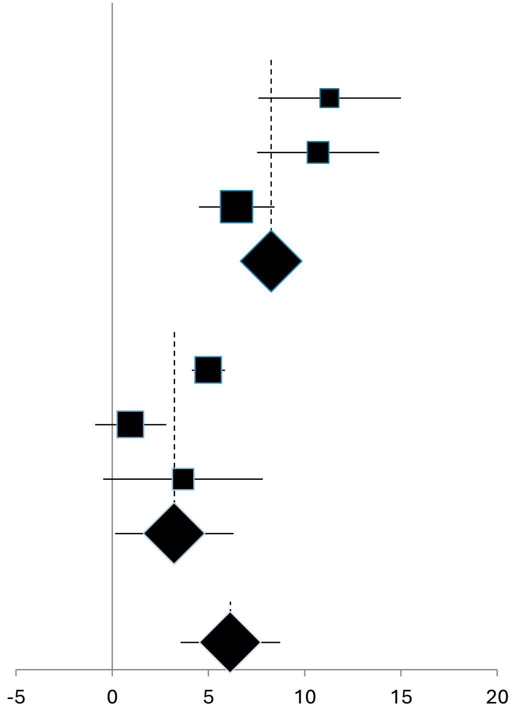

WMD – weighted mean difference; MD – mean difference; CI – confidence interval; N/A – not applicable; P – periodontitis; C – control

Figure S7 . Prediction line determined by meta-regression for difference in percentage of females (periodontitis group versus control group) as an independent variable

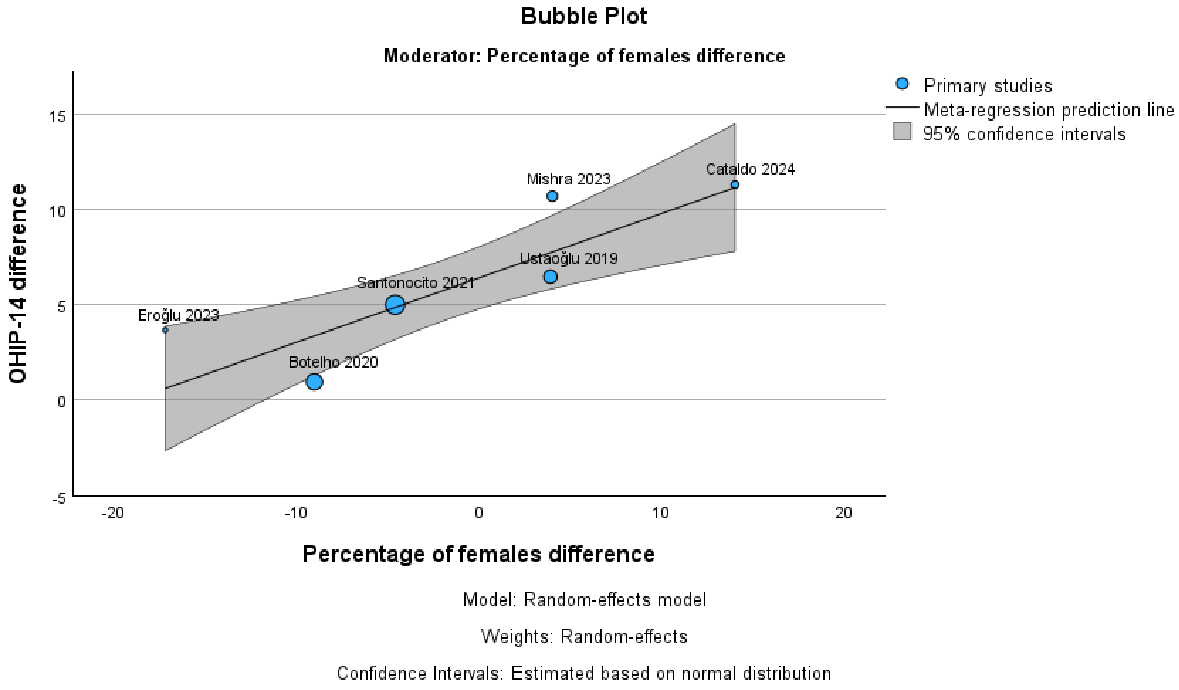

Figure S8 . Prediction line determined by meta-regression for percentage of females in the sample as an independent variable

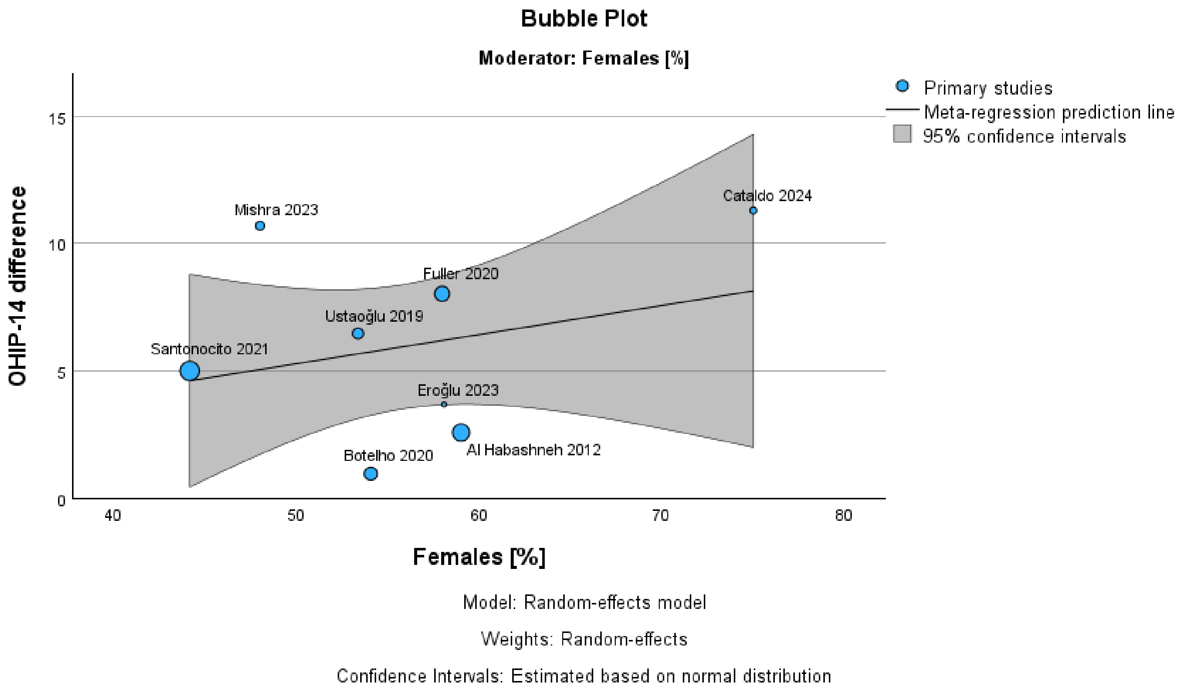

Figure S9 . Weighted mean difference in severity of impacts (mean OHIP-14 total score) in periodontitis patients compared to control subjects, meta-analyses in subgroups of studies by the difference in mean PPD between P and C groups; only studies with available mean PPD for P and C groups were included

Study	PPD difference (P vs C) [mm]	N Periodontitis	N Controls	Weight [%]	MD (95% CI)
<u>Mean PPD in P group higher by ≥2 mm</u>					
Mishra 2023	3,0	50	50	23	10.70 (7.53; 13.87)
Cataldo 2024	2,6	50	50	16.9	11.30 (7.59; 15.01)
Ustaoğlu 2019	2,0	114	109	60.1	6.47 (4.51; 8.43)
**WMD (95% CI) [fixed; p = 0.0179, I^2 = 75.2%]**		**214**	**209**	**n/a**	**8.26 (6.74; 9.78),** p < 0.0001
<u>Mean PPD in P group higher by <2 mm</u>					
Dikilitaş 2024 (P vs pooled C)	1,7	36	130	47.7	7.78 (6.33; 9.23)
Santonocito 2021	1,3	55	56	52.3	5.00 (4.14; 5.86)
**WMD (95% CI) [random; p = 0.0012, I^2 = 90.4%]**		**91**	**186**	**n/a**	**6.33 (3.61; 9.05),** p < 0.0001
<u>Total</u>					
**WMD (95% CI) [random; p < 0.0001, I^2 = 85.3%]**		**305**	**395**	**n/a**	**7.80 (5.67; 9.93), p < 0.0001**

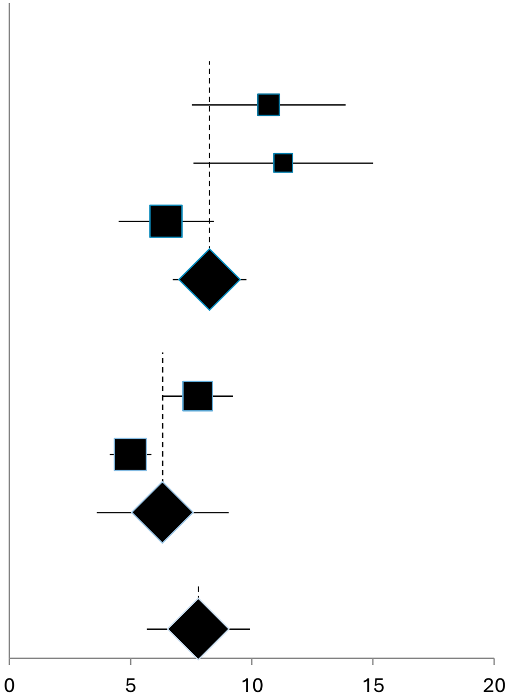

WMD – weighted mean difference; MD – mean difference; CI – confidence interval; N/A – not applicable; P – periodontitis; C – control

Figure S10 . Prediction line determined by meta-regression for difference in mean probing pocket depth (periodontitis group versus control group) as an independent variable

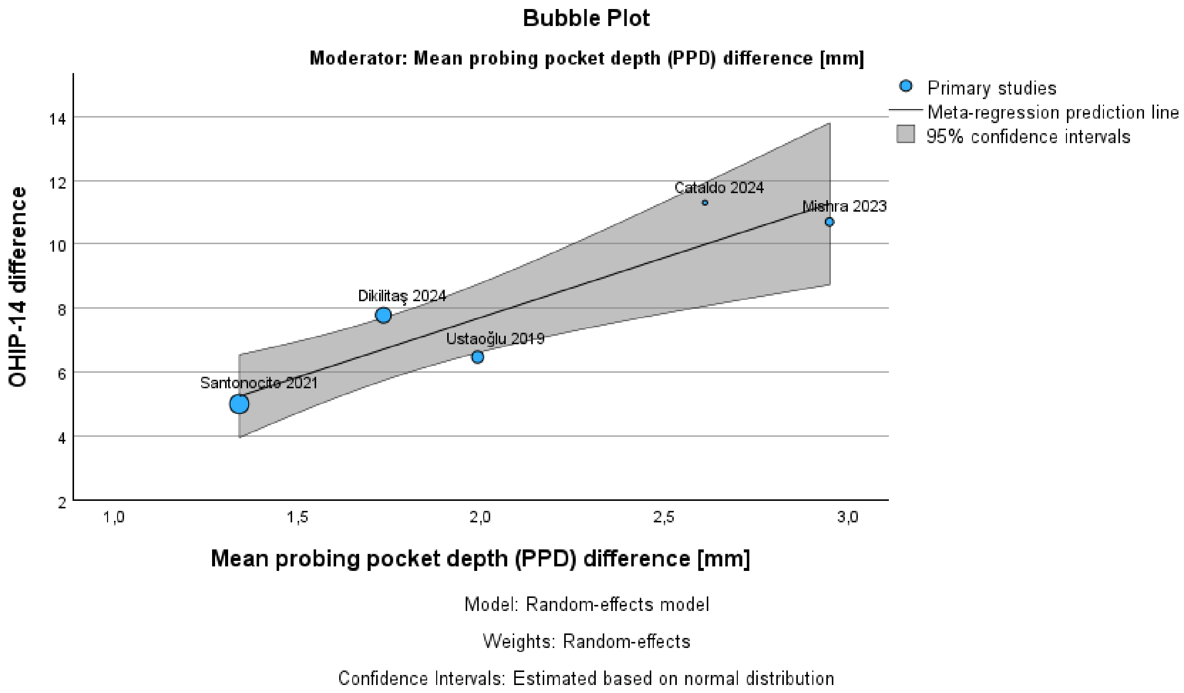

Figure S11 . Prediction line determined by meta-regression for mean probing pocket depth in the sample as an independent variable

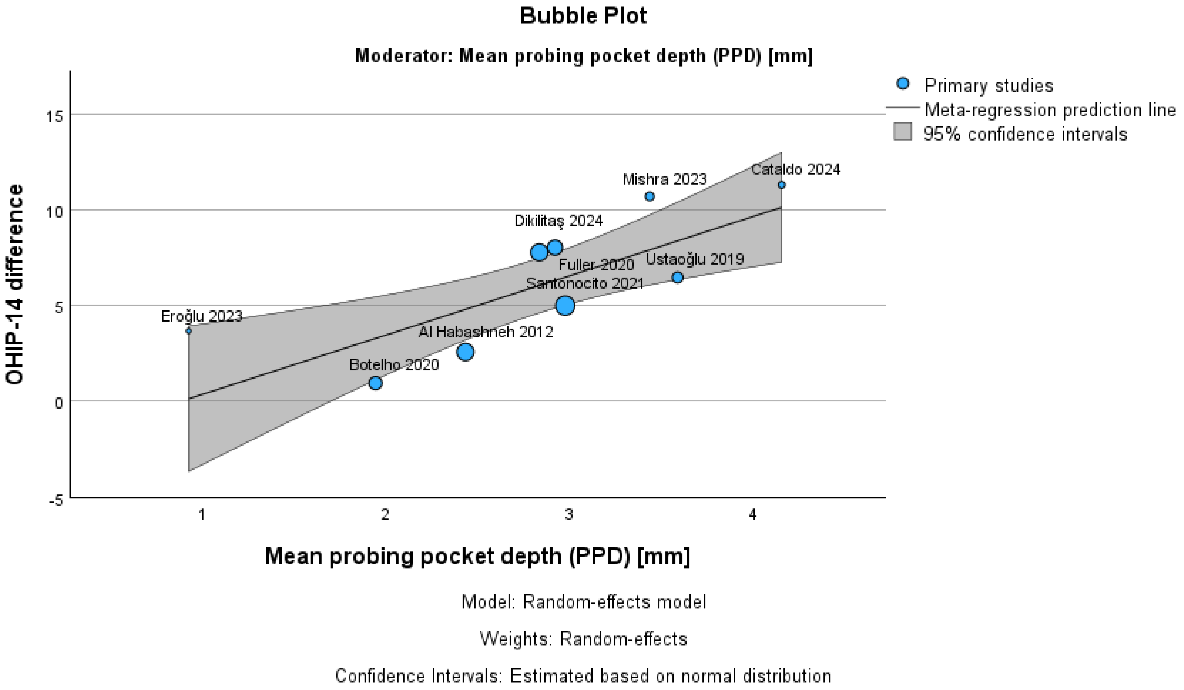

Table S8. PRISMA 2020 Checklist (25)

Section and Topic	Item #	Checklist item	Location where item is reported
**TITLE**			
Title	1	Identify the report as a systematic review.	Title, keywords
**ABSTRACT**			
Abstract	2	See the PRISMA 2020 for Abstracts checklist.	Abstract
**INTRODUCTION**			
Rationale	3	Describe the rationale for the review in the context of existing knowledge.	Introduction, paragraphs 1–3
Objectives	4	Provide an explicit statement of the objective(s) or question(s) the review addresses.	Introduction, paragraph 4
**METHODS**			
Eligibility criteria	5	Specify the inclusion and exclusion criteria for the review and how studies were grouped for the syntheses.	Search strategy and selection criteria, Data analysis, Supplementary materials
Information sources	6	Specify all databases, registers, websites, organisations, reference lists and other sources searched or consulted to identify studies. Specify the date when each source was last searched or consulted.	Search strategy and selection criteria, paragraph 1
Search strategy	7	Present the full search strategies for all databases, registers and websites, including any filters and limits used.	Supplementary materials, tables S1-S3
Selection process	8	Specify the methods used to decide whether a study met the inclusion criteria of the review, including how many reviewers screened each record and each report retrieved, whether they worked independently, and if applicable, details of automation tools used in the process.	Data analysis, paragraph 1
Data collection process	9	Specify the methods used to collect data from reports, including how many reviewers collected data from each report, whether they worked independently, any processes for obtaining or confirming data from study investigators, and if applicable, details of automation tools used in the process.	Data analysis, paragraph 1
Data items	10a	List and define all outcomes for which data were sought. Specify whether all results that were compatible with each outcome domain in each study were sought (e.g. for all measures, time points, analyses), and if not, the methods used to decide which results to collect.	Search strategy and selection criteria, paragraph 3 and Supplementary materials, Table S4

Section and Topic	Item #	Checklist item	Location where item is reported
	10b	List and define all other variables for which data were sought (e.g. participant and intervention characteristics, funding sources). Describe any assumptions made about any missing or unclear information.	Data analysis, paragraph 1
Study risk of bias assessment	11	Specify the methods used to assess risk of bias in the included studies, including details of the tool(s) used, how many reviewers assessed each study and whether they worked independently, and if applicable, details of automation tools used in the process.	Data analysis, paragraph 2, and Supplementary materials, Table S6
Effect measures	12	Specify for each outcome the effect measure(s) (e.g. risk ratio, mean difference) used in the synthesis or presentation of results.	Data analysis, paragraph 3
Synthesis methods	13a	Describe the processes used to decide which studies were eligible for each synthesis (e.g. tabulating the study intervention characteristics and comparing against the planned groups for each synthesis (item #5)).	Data analysis, paragraph 3
	13b	Describe any methods required to prepare the data for presentation or synthesis, such as handling of missing summary statistics, or data conversions.	Data analysis, paragraph 1
	13c	Describe any methods used to tabulate or visually display results of individual studies and syntheses.	Data analysis, paragraph 3
	13d	Describe any methods used to synthesize results and provide a rationale for the choice(s). If meta-analysis was performed, describe the model(s), method(s) to identify the presence and extent of statistical heterogeneity, and software package(s) used.	Data analysis, paragraph 3
	13e	Describe any methods used to explore possible causes of heterogeneity among study results (e.g. subgroup analysis, meta-regression).	Data analysis, paragraph 3
	13f	Describe any sensitivity analyses conducted to assess robustness of the synthesized results.	Data analysis (subgroup analyses, meta-regression)
Reporting bias assessment	14	Describe any methods used to assess risk of bias due to missing results in a synthesis (arising from reporting biases).	Data analysis, paragraph 2
Certainty assessment	15	Describe any methods used to assess certainty (or confidence) in the body of evidence for an outcome.	Not applicable
**RESULTS**			
Study selection	16a	Describe the results of the search and selection process, from the number of records identified in the search to the number of studies included in the review, ideally using a flow diagram.	Included studies, paragraph 1 and Supplementary materials, Figure S1
	16b	Cite studies that might appear to meet the inclusion criteria, but which were excluded, and explain why they were excluded.	Not applicable
Study characteristics	17	Cite each included study and present its characteristics.	Included studies, paragraph 2–3, and

Section and Topic	Item #	Checklist item	Location where item is reported
			Table 1, Table 2
Risk of bias in studies	18	Present assessments of risk of bias for each included study.	Included studies, paragraph 6, Table 1 and Supplementary materials, Table S5, Figure S2
Results of individual studies	19	For all outcomes, present, for each study: (a) summary statistics for each group (where appropriate) and (b) an effect estimate and its precision (e.g. confidence/credible interval), ideally using structured tables or plots.	Table 3, Figures 1–5 and Supplementary materials, Figures S4, S7, S10
Results of syntheses	20a	For each synthesis, briefly summarise the characteristics and risk of bias among contributing studies.	Not applicable
	20b	Present results of all statistical syntheses conducted. If meta-analysis was done, present for each the summary estimate and its precision (e.g. confidence/credible interval) and measures of statistical heterogeneity. If comparing groups, describe the direction of the effect.	Results of the meta-analyses, paragraphs 1–5, Figures 1–5 and Supplementary materials, Figures S4–S12
	20c	Present results of all investigations of possible causes of heterogeneity among study results.	Results of the meta-analyses, paragraphs 1–5, Figures 2–5 and Supplementary materials, Figures S4–S12
	20d	Present results of all sensitivity analyses conducted to assess the robustness of the synthesized results.	Results of the meta-analyses (subgroup analyses, meta-regression)
Reporting biases	21	Present assessments of risk of bias due to missing results (arising from reporting biases) for each synthesis assessed.	Presented for the main synthesis (Included studies, paragraph 9 and Supplementary materials, Figure S3)

Section and Topic	Item #	Checklist item	Location where item is reported
Certainty of evidence	22	Present assessments of certainty (or confidence) in the body of evidence for each outcome assessed.	Not applicable
**DISCUSSION**			
Discussion	23a	Provide a general interpretation of the results in the context of other evidence.	Discussion, paragraphs 1–2, 4–5, 7
	23b	Discuss any limitations of the evidence included in the review.	Discussion, paragraphs 3, 10
	23c	Discuss any limitations of the review processes used.	Discussion, paragraph 10
	23d	Discuss implications of the results for practice, policy, and future research.	Conclusions
**OTHER INFORMATION**			
Registration and protocol	24a	Provide registration information for the review, including register name and registration number, or state that the review was not registered.	Not registered
	24b	Indicate where the review protocol can be accessed, or state that a protocol was not prepared.	Internal protocol, available upon request from the authors
	24c	Describe and explain any amendments to information provided at registration or in the protocol.	Not applicable
Support	25	Describe sources of financial or non-financial support for the review, and the role of the funders or sponsors in the review.	Funding statement
Competing interests	26	Declare any competing interests of review authors.	Conflict of interest statement
Availability of data, code and other materials	27	Report which of the following are publicly available and where they can be found: template data collection forms; data extracted from included studies; data used for all analyses; analytic code; any other materials used in the review.	Available upon request from the authors

This work is licensed under CC BY 4.0. To view a copy of this license, visit https://creativecommons.org/licenses/by/4.0/

References

1. NHLBI. *Quality Assessment Tool for Observational Cohort and Cross-Sectional Studies. Last Updated: July, 2021*: The National Heart, Lung, and Blood Institute. Available from: https://www.nhlbi.nih.gov/health-topics/study-quality-assessment-tools.
2. Al Habashneh R, Khader YS, Salameh S. Use of the Arabic Version of Oral Health Impact Profile-14 to Evaluate the Impact of Periodontal Disease on Oral Health-Related Quality of Life among Jordanian Adults. *J Oral Sci* (2012) 54(1):113-20. doi: 10.2334/josnusd.54.113.
3. Botelho J, Machado V, Proenca L, Oliveira MJ, Cavacas MA, Amaro L, et al. Perceived Xerostomia, Stress and Periodontal Status Impact on Elderly Oral Health-Related Quality of Life: Findings from a Cross-Sectional Survey. *BMC Oral Health* (2020) 20(1):199. doi: 10.1186/s12903-020-01183-7.
4. Cataldo D, Mourao LC, Goncalves LS, Canabarro A. Association of Anxiety, Age and Oral Health-Related Quality of Life with Periodontitis: A Case-Control Study. *Int J Dent Hyg* (2024) 22(3):540-6. doi: 10.1111/idh.12687.
5. Dikilitas A, Karaaslan F, Evirgen S, Ertugrul AS, Kamburoglu K. Evaluation of the Effects of Periodontal Status According to 2017 Classifications on Oral Health-Related Quality of Life and Quality of Life-Related to General Health: A Cross-Sectional Study. *Int J Dent Hyg* (2024) 22(3):672-80. doi: 10.1111/idh.12753.
6. Eroğlu ZT, Şen DÖ, Yarkac FU, Altiparmak F. The Association between Sleep Quality, Fatigue and Periodontal Status: A Pilot Study. *Odovtos - International Journal of Dental Sciences* (2023) 25(3):99-117. doi: 10.15517/ijds.2023.54866.
7. Fuller J, Donos N, Suvan J, Tsakos G, Nibali L. Association of Oral Health-Related Quality of Life Measures with Aggressive and Chronic Periodontitis. *J Periodontal Res* (2020) 55(4):574-80. doi: 10.1111/jre.12745.
8. Mishra S, Johnson L, Kaushal L, Upadhyay P. Impact of Periodontitis on Oral Health-Related Quality of Life of Patients with Psoriatic Arthritis. *Spec Care Dentist* (2024) 44(3):893-902. doi: 10.1111/scd.12938.
9. Santonocito S, Polizzi A, Ronsivalle V, Palazzo G, Sicari F, Isola G. Impact of Periodontitis on Systemic Anxiety and Oral Health Quality of Life. *Mediterranean Journal of Clinical Psychology* (2021) 9:1-16. doi: 10.13129/2282-1619/mjcp-3064.
10. Ustaoglu G, Goller Bulut D, Gumus KC, Ankarali H. Evaluation of the Effects of Different Forms of Periodontal Diseases on Quality of Life with Ohip-14 and Sf-36 Questionnaires: A Cross-Sectional Study. *Int J Dent Hyg* (2019) 17(4):343-9. doi: 10.1111/idh.12409.
11. Botelho J, Machado V, Proenca L, Alves R, Cavacas MA, Amaro L, et al. Study of Periodontal Health in Almada-Seixal (Sophias): A Cross-Sectional Study in the Lisbon Metropolitan Area. *Sci Rep* (2019) 9(1):15538. doi: 10.1038/s41598-019-52116-6.
12. Armitage GC. Development of a Classification System for Periodontal Diseases and Conditions. *Ann Periodontol* (1999) 4(1):1-6. doi: 10.1902/annals.1999.4.1.1.
13. Afonso A, Silva I, Meneses R, Frias-Bulhosa J, Afonso A. Oral Health-Related Quality of Life: Portuguese Linguistic and Cultural Adaptation of Ohip-14. *Psicologia, Saúde & Doença* (2017) 18:374-88. doi: 10.15309/17psd180208.
14. Oliveira BH, Nadanovsky P. Psychometric Properties of the Brazilian Version of the Oral Health Impact Profile-Short Form. *Community Dent Oral Epidemiol* (2005) 33(4):307-14. doi: 10.1111/j.1600-0528.2005.00225.x.
15. Mumcu G, Inanc N, Ergun T, Ikiz K, Gunes M, Islek U, et al. Oral Health Related Quality of Life Is Affected by Disease Activity in Behcet's Disease. *Oral Dis* (2006) 12(2):145-51. doi: 10.1111/j.1601-0825.2005.01173.x.

16. Tonetti MS, Greenwell H, Kornman KS. Staging and Grading of Periodontitis: Framework and Proposal of a New Classification and Case Definition. *J Periodontol* (2018) 89 Suppl 1:S159-S72. doi: 10.1002/JPER.18-0006.
17. Chapple ILC, Mealey BL, Van Dyke TE, Bartold PM, Dommisch H, Eickholz P, et al. Periodontal Health and Gingival Diseases and Conditions on an Intact and a Reduced Periodontium: Consensus Report of Workgroup 1 of the 2017 World Workshop on the Classification of Periodontal and Peri-Implant Diseases and Conditions. *J Periodontol* (2018) 89 Suppl 1:S74-S84. doi: 10.1002/JPER.17-0719.
18. Papapanou PN, Sanz M, Buduneli N, Dietrich T, Feres M, Fine DH, et al. Periodontitis: Consensus Report of Workgroup 2 of the 2017 World Workshop on the Classification of Periodontal and Peri-Implant Diseases and Conditions. *J Periodontol* (2018) 89 Suppl 1:S173-S82. doi: 10.1002/JPER.17-0721.
19. Slade GD. Derivation and Validation of a Short-Form Oral Health Impact Profile. *Community Dent Oral Epidemiol* (1997) 25(4):284-90. doi: 10.1111/j.1600-0528.1997.tb00941.x.
20. Eke PI, Page RC, Wei L, Thornton-Evans G, Genco RJ. Update of the Case Definitions for Population-Based Surveillance of Periodontitis. *J Periodontol* (2012) 83(12):1449-54. doi: 10.1902/jop.2012.110664.
21. Page RC, Eke PI. Case Definitions for Use in Population-Based Surveillance of Periodontitis. *J Periodontol* (2007) 78(7 Suppl):1387-99. doi: 10.1902/jop.2007.060264.
22. Deshpande NC, Nawathe AA. Translation and Validation of Hindi Version of Oral Health Impact Profile-14. *J Indian Soc Periodontol* (2015) 19(2):208-10. doi: 10.4103/0972-124X.145806.
23. Corridore D, Campus G, Guerra F, Ripari F, Sale S, Ottolenghi L. Validation of the Italian Version of the Oral Health Impact Profile-14 (Iohip-14). *Ann Stomatol (Roma)* (2013) 4(3-4):239-43. Epub 20140204.
24. McGuinness LA, Higgins JPT. Risk-of-Bias Visualization (Robvis): An R Package and Shiny Web App for Visualizing Risk-of-Bias Assessments. *Res Synth Methods* (2021) 12(1):55-61. doi: 10.1002/jrsm.1411.
25. Page MJ, McKenzie JE, Bossuyt PM, Boutron I, Hoffmann TC, Mulrow CD, et al. The Prisma 2020 Statement: An Updated Guideline for Reporting Systematic Reviews. *BMJ* (2021) 372:n71. doi: 10.1136/bmj.n71.
